# Tryptophan-Enriched *Lactobacillus rhamnosus* GG-derived Nanovesicles Promote Alveolar Bone Regeneration through Macrophage Fatty Acid Oxidation

**DOI:** 10.34133/bmr.0370

**Published:** 2026-07-17

**Authors:** Zhendong Huang, Yuqi Liu, Qiudong Yang, Jiayi Ding, Zhengkun Yang, Heyu Liu, Xin Huang, Xiaoxuan Wang, Li Ma, Zhengguo Cao

**Affiliations:** ^1^ State Key Laboratory of Oral & Maxillofacial Reconstruction and Regeneration, Key Laboratory of Oral Biomedicine Ministry of Education, Hubei Key Laboratory of Stomatology, School & Hospital of Stomatology, Wuhan University, Wuhan, China.; ^2^ Department of Periodontology, School & Hospital of Stomatology, Wuhan University, Wuhan, China.

## Abstract

Inflammatory bone loss represents a major clinical challenge, leading to irreversible tissue damage and impaired function. Probiotic-derived nanovesicles show immense potential as novel cell-free nanomedicines; however, the lack of clarity regarding their precise mechanism of action in bone tissue regeneration restricts their clinical application. This study utilized a ligature-induced periodontitis mouse model (in vivo) and in vitro models, including macrophage functional assays and macrophage–osteoblast co-culture systems, to investigate the therapeutic effects and mechanism of *Lactobacillus rhamnosus* GG-derived extracellular vesicles (LEVs). In the periodontitis mouse model, LEVs effectively mitigated inflammatory infiltration and promoted alveolar bone regeneration. In vitro studies demonstrated that LEVs enhance macrophage polarization toward a reparative (M2) phenotype. Mechanistically, we identify LEVs as bioactive nanocarriers that deliver tryptophan metabolites. Upon internalization by macrophages, these metabolites trigger a critical metabolic and phenotypic shift by activating the aryl hydrocarbon receptor (AhR). Further research revealed that this effect is mediated by the AhR/NAD(P)H:quinone oxidoreductase 1 (NQO1)/carnitine palmitoyltransferase 1A (CPT1A) signaling axis: AhR transcriptionally up-regulates NQO1, which critically inhibits the 26S proteasome-mediated degradation of CPT1A. The resulting sustained CPT1A expression dramatically boosts fatty acid oxidation, which is essential for driving the reparative macrophage phenotype. These findings highlight the critical role and molecular delivery mechanisms of probiotic-derived nanovesicles in ameliorating inflammatory bone loss via immunometabolic reprogramming, thereby providing new targets and a theoretical basis for their application as nanocarriers in regenerative biomaterials.

## Introduction

Inflammatory bone loss represents a significant global health challenge, fundamentally characterized by immune cell dysfunction that leads to impaired osteoblast function and enhanced osteoclast activity, ultimately resulting in progressive bone loss [1,2]. This pathological process is prevalent in a wide range of chronic inflammatory diseases, such as rheumatoid arthritis, osteoarthritis, and periodontitis, severely affecting patients’ quality of life and physiological function. In the craniofacial region, periodontitis is a typical disease of inflammatory bone loss, presenting as progressive alveolar bone resorption [3]. Periodontitis, as a highly prevalent chronic inflammatory disease, is a primary cause of progressive destruction of alveolar bone and the leading cause of tooth loss in adults [4]. It not only impairs masticatory function and oral aesthetics but is also closely associated with various systemic diseases, including cardiovascular disease, diabetes, and obesity [5]. Traditional treatments mainly focus on controlling local inflammation, but the regenerative capacity of severely damaged bone tissue is very limited. Therefore, in the field of regenerative medicine, there is an urgent need to develop novel therapeutic strategies that can effectively promote bone regeneration in an inflammatory environment, thereby reversing the disease process.

The probiotic *Lactobacillus rhamnosus* GG (*LGG*) has garnered significant attention due to its potent immunomodulatory and anti-inflammatory properties [6]; its established GRAS (generally recognized as safe) status and proven clinical efficacy in modulating dental plaque and gingival inflammation further highlight its therapeutic potential in oral disease therapy [7,8]. Crucially, as a prototypical gram-positive bacterium, *LGG* is inherently devoid of lipopolysaccharides (LPSs), a key attribute that effectively circumvents the interference or exacerbation of the localized inflammatory microenvironment by exogenous endotoxins. Nevertheless, considering that the bioactivity of *LGG* is often compromised by the inactivation processes typically required for clinical application, probiotic-derived bacterial extracellular vesicles (BEVs) are being extensively investigated as a promising alternative. Compared to mammalian-cell-derived exosomes [9], BEVs offer lower production costs, higher yields [10], and fewer immunosuppressive components [11], demonstrating superior translational advantages. As bioactive nanocarriers, *LGG*-derived extracellular vesicles (LEVs) have been shown to exert immunomodulatory effects by enhancing macrophage phagocytosis and mediate beneficial functions by delivering effector molecules inherited from their parent bacteria [12–16].

Although LEVs show broad application prospects in various diseases, their role and underlying molecular mechanisms in inflammatory bone repair remain limited. A clear elucidation of the underlying molecular mechanisms would significantly facilitate their clinical application and translation in regenerative therapies [17]. The development and progression of inflammation are closely linked to the local microenvironment, within which macrophages, acting as key regulators of the inflammatory response, play a crucial role in bone destruction. Emerging evidence in the field of immunometabolism indicates that macrophage functional plasticity is fundamentally governed by distinct metabolic programs. Specifically, pro-inflammatory (M1) polarization is primarily driven by enhanced glycolysis, whereas the transition toward a pro-resolving and reparative (M2) phenotype requires a metabolic shift toward mitochondrial oxidative phosphorylation and fatty acid oxidation (FAO). In the context of periodontitis, rebalancing these metabolic pathways is essential for resolving chronic inflammation and initiating tissue repair. While LEVs are known to be internalized by macrophages [13,18,19], whether these nanovesicles can shift the local microenvironment from osteolysis to regeneration, and whether they regulate macrophage polarization by reprogramming macrophage metabolism, remains unclear.

Our study aims to fill this critical research gap and thoroughly investigate the therapeutic potential of LEVs in inflammatory bone regeneration and their underlying molecular mechanisms. We verified that LEVs can effectively promote alveolar bone regeneration in a periodontitis model through both in vitro and in vivo experiments. More importantly, our study detailed the upstream molecular mechanism by which LEVs activate the aryl hydrocarbon receptor (AhR) to transcriptionally up-regulate NAD(P)H:quinone oxidoreductase 1 (NQO1), which in turn inhibits the degradation of carnitine palmitoyltransferase 1A (CPT1A) mediated by the 26S proteasome, ultimately enhancing macrophage FAO levels. This upstream mechanism effectively promotes macrophage polarization toward a reparative phenotype, leading to effective bone repair. These findings establish LEVs as a novel, mechanistically defined nanomedicine platform for inflammatory bone loss and provide new insights and strategies for leveraging bacterial nanocarriers to achieve tissue regeneration through metabolic reprogramming.

## Materials and Methods

### *LGG* strain culture and LEV isolation, identification, and characterization

*LGG* (ATCC 53103) was purchased from Guangdong Microbial Culture Collection Center. *LGG* was inoculated into MRS medium (LuQiao, Beijing, China) and grown anaerobically. In a sterile cabinet, the culture medium was centrifuged at 10,000 × *g* for 30 min at 4 °C to remove bacteria. The supernatant was collected and filtered through a 0.22-μm sterile filter membrane (Millipore) to ensure the removal of all remaining bacteria and larger debris. The filtered supernatant was then concentrated using an ultrafiltration membrane (Millipore Pellicon XL, 100-kDa cutoff). Subsequently, the concentrated solution was aliquoted into 70-ml ultracentrifuge tubes and centrifuged at 170,000 × *g* (approximately 38,200 rpm) for 2 h at 4 °C in an ultracentrifuge (Optima XPN-100, Beckman Coulter) equipped with a Type 45 Ti fixed-angle rotor to obtain LEVs. The LEV pellet was resuspended in sterile phosphate-buffered saline (PBS) and centrifuged again for further purification. Finally, the purified LEV suspension was aliquoted and stored at −80 °C until use. To verify that the biological effects were specific to LEVs and not derived from culture medium components, a processed medium (PM) control was included. Sterile, uninoculated MRS broth was subjected to the identical isolation and purification procedure as that described for LEVs. The resulting pellet area in the PM group was resuspended in a volume of PBS equal to that used for LEV samples. This PM control was used in subsequent Western blot and functional assays to ensure the exclusion of any confounding medium-derived background. LEVs were characterized by transmission electron microscopy, nanoparticle tracking analysis (NTA), and Western blot analysis for the gram-positive bacterial marker lipoteichoic acid (LTA). Transmission electron microscopy (Hitachi) was used to analyze the morphology and ultrastructure of the LEVs. NTA (ZetaView) was employed to determine the size distribution of the LEVs. Western blot analysis was performed to determine the protein level of the gram-positive bacterial marker LTA. The presumed concentration of LEVs was determined using a bicinchoninic acid assay kit (Beyotime, no. P0010).

### Antibodies and drugs

Mouse anti-LTA (no. HM5018) was purchased from Hycult Biotech. Mouse anti-CPT1A (no. 66039-3-Ig), rabbit anti-arginase-1 (anti-ARG1; no. 166129-1-Ig), rabbit anti-AhR (no. 28727-1-AP), rabbit anti-inducible nitric oxide synthase (anti-iNOS; no. 18985-1-AP), rabbit anti-osteocalcin (anti-OCN; no. 23418-1-AP), rabbit anti-S100A8 (no. 15792-1-AP), rabbit anti-26S proteasome (no. 14748-1-AP), mouse anti-TATA-binding protein (no. 66166-1-Ig), rabbit anti-20S proteasome (no. 14544-1-AP), rabbit anti-Flag (no. 20543-1-AP), and mouse anti-β-actin (no. 66009-1-Ig) were purchased from Proteintech. Rabbit anti-acyl-CoA synthetase long-chain family member 1 (anti-ACSL1; no. A24751), rabbit anti-hydroxyacyl-CoA dehydrogenase trifunctional multienzyme complex subunit alpha (anti-HADHA; no. 5346), rabbit anti-CD163 (no. A26411PM), rabbit anti-acyl-CoA dehydrogenase medium chain (anti-ACADM; no. A1873), and rabbit anti-NQO1 (no. 23486) were purchased from ABclonal. Rat anti-F4/80 (no. ab16911), mouse anti-CD86 (no. ab220188), rabbit anti-Runt-related transcription factor 2 (anti-Runx2; no. ab236639), and rabbit anti-Osterix (no. ab22552) were purchased from Abcam. Rabbit anti-CD206 (no. 24595) was purchased from Cell Signaling Technology. Mouse anti-interleukin-10 (anti-IL-10; no. MA5-23796) was purchased from Thermo Fisher Scientific. Rabbit anti-interleukin-6 (anti-IL-6; no. DF6087) and rabbit anti-bone sialoprotein (anti-BSP; no. DF7738) were purchased from Affinity. Anti-ubiquitin (no. PTM-1106RM) was purchased from PTM Bio. *Porphyromonas gingivalis* was derived from ATCC 33277; LPS from *P. gingivalis* (*Pg*LPS; no. tlrl-pglps) was purchased from InvivoGen. Dimethyl sulfoxide (no. A36720100) was purchased from AppliChem Darmstadt. Cycloheximide (CHX; no. HY-1232), bafilomycin A1 (BFA; no. HY-100558), MG-132 (no. HY-13259), PYR-41 (no. HY-13296), CH-223191 (no. HY-12684), Dynasore (no. HY-15304), nicotinamide adenine dinucleotide + hydrogen (NADH; no. HY-F0001), dicumarol (no. HY-N0645), and etomoxir (no. HY-50202) were purchased from MedChemExpress. The recombinant mouse IL-10 (rmIL-10) protein was purchased from ABclonal Technology (no. RP01465).

### Periodontitis modeling, etomoxir administration, and LEV treatment in mice

Male C57BL/6 mice (8-week-old) were provided by the Laboratory Animal Research Center of Hubei Provincial Center for Disease Control and Prevention. To establish the periodontitis model, a 5-0 silk ligature was tied around the maxillary second molar of each mouse under intraperitoneal anesthesia on day 0. Crucially, the ligatures remained in place for 4 d without any intervention to allow for the robust establishment of early alveolar bone resorption. Following a 4-d disease establishment phase, therapeutic interventions were initiated. The etomoxir group received daily intraperitoneal injections of etomoxir (3 mg/kg) for 3 consecutive days starting from day 4. The LEV group received local microinjections of LEVs (50 μg) into the gingival tissue every 3 d, strictly initiated on day 4. The LEV dosage was adopted from established protocols for LEVs in previous regenerative therapeutic model research [20,21]. This dose has been proven effective for modulating macrophage polarization and promoting tissue repair without inducing local toxicity. All mice were euthanized on day 12 postligation. Animal protocols were approved by the Ethics Committee of the School and Hospital of Stomatology, Wuhan University (S07925060D).

### Micro-CT analysis

After euthanasia, the mice were dissected, and the maxillae were isolated. The maxillae were then fixed in 4% paraformaldehyde (PFA) for 24 h. Subsequently, the samples were scanned using a Quantum GX micro-computed tomography (micro-CT) scanner (PerkinElmer, Inc., Waltham, MA, USA) at a voltage of 100 kV, a current of 200 μA, and a pixel size of 10 μm. The CTAn and ANT software were used for the 3-dimensional reconstruction and quantitative analysis of the distance from the cementoenamel junction (CEJ) to the alveolar bone crest (ABC) (CEJ–ABC) and the bone volume/total volume (BV/TV) ratio.

### Histopathology and immunofluorescence

After decalcification, the maxillae were embedded in paraffin and sectioned at a thickness of 5 μm. The sections were stained with hematoxylin and eosin (H&E) to assess the integrity of the CEJ and the infiltration of inflammatory cells in the periodontal tissue. In addition, tartrate-resistant acid phosphatase (TRAP) staining was performed to identify osteoclasts, and alkaline phosphatase (ALP) staining was used to identify osteoblasts.

Subsequently, the sections were stained using a Masson’s trichrome staining kit (Solarbio, Beijing, China) according to the manufacturer’s protocol. Briefly, sections were initially stained with Weigert’s iron hematoxylin for 5 to 10 min, differentiated, and then subjected to Ponceau–acid fuchsin staining for 5 to 10 min. After washing, the sections were differentiated in phosphomolybdic–phosphotungstic acid solution for 1 to 3 min and subsequently stained with aniline blue solution for 5 to 10 min. Following rapid dehydration with ethanol and clearing with xylene, the slides were mounted with neutral resin.

Gingival tissues were processed for frozen tissue sectioning, typically by embedding them in optimal cutting temperature compound and sectioning at a thickness of 5 μm. Finally, cell nuclei were counterstained with 4′,6-diamidino-2-phenylindole (DAPI), and images were captured using a Leica STELLARIS fluorescence microscope.

### Cell culture

The mouse macrophage cell line RAW264.7 (Research Resource Identifier [RRID]: CVCL_0493), human monocytic cell line THP-1 (RRID: CVCL_0006), and mouse preosteoblast cell line MC3T3-E1 (RRID: CVCL_5437) were all purchased from Procell Life Science & Technology Co., Ltd. (Wuhan, China) with catalog numbers CL-0190, CL-0233, and CL-0378, respectively. Mycoplasma contamination was routinely tested using a mycoplasma detection kit (Solarbio, no. CA1080), and all cell lines were confirmed to be mycoplasma-free. RAW264.7 and MC3T3-E1 cells were cultured in Dulbecco’s modified Eagle medium (DMEM). THP-1 cells were seeded into 6-well plates and induced into M0 macrophages by 320 nM phorbol 12-myristate 13-acetate (HY-18739, MedChemExpress) in serum-free RPMI 1640 medium for 48 h.

The isolation and culture of periodontal ligament stem cells (PDLSCs) were performed using an enzymatic digestion method. Periodontal ligament tissues were carefully scraped from healthy extracted teeth under sterile conditions. The minced tissues were then placed in a culture medium containing type I collagenase and dispase for digestion at 37 °C. After digestion, the cell suspension was passed through a 40-μm cell strainer to remove any undigested tissue fragments, and the cells were collected by centrifugation. The cell pellet was subsequently resuspended in alpha minimum essential medium (α-MEM) supplemented with 20% fetal bovine serum (FBS) and 1% penicillin–streptomycin and then seeded into a culture dish. The cells were incubated in a humidified incubator at 37 °C with 5% CO_2_, and the medium was replaced every 2 to 3 d until the cells reached 80% to 90% confluency.

Bone-marrow-derived macrophages (BMDMs) were isolated and generated from 8-week-old mice. After collecting the tibias and femurs, bone marrow cells were flushed out using α-MEM supplemented with 2% FBS. Following red blood cell lysis, the BMDM cells were cultured for 3 d with macrophage colony-stimulating factor (ABclonal, China) in α-MEM containing 10% FBS to obtain BMDMs.

The osteogenic induction medium for PDLSCs and MC3T3-E1 was prepared by adding additional osteogenic induction components to a conventional complete culture. Osteogenic medium (OM) with DMEM or α-MEM, 10% FBS, 10 mM sodium β-glycerophosphate, 10 nM dexamethasone, and 50 μg/ml ascorbic acid was used to stimulate the formation of osteogenic differentiation.

### Macrophage-derived conditioned-medium-induced osteogenesis

The construction of the co-culture system and the preparation of the conditioned medium (CM) referred to previous studies [22,23]. To prepare the CM, RAW264.7 or THP-1 macrophages were first incubated with LEVs or LPS for 24 h for stimulation. Subsequently, the culture supernatant was removed, and the cells were washed with fresh, serum-free medium to remove residual stimuli. Fresh serum-free medium was then added, and the cells were cultured for an additional 12 h. Finally, the culture supernatant was collected and centrifuged to remove cell debris and other residues, and the resulting supernatant was used as subsequent osteogenic culture of MC3T3-E1 or PDLSCs.

MC3T3-E1 or PDLSCs were seeded into culture plates. Once the cells had fully adhered, the regular medium was removed and replaced with a mixed osteogenic differentiation medium. This medium was composed of a suitable ratio of osteogenic induction medium and the prepared CM. After 2 weeks of osteogenic induction, the cells in the culture plates were fixed with 4% PFA and then stained using an ALP staining kit (no. C3206, Beyotime, Shanghai, China). In addition, after 3 weeks of osteogenic induction, the cells were fixed with 4% PFA and stained with 1% Alizarin Red S (ARS; no. ALIR-10001, Saiye Bio, Guangzhou).

To evaluate whether IL-10 directly and independently stimulates osteoblast differentiation, a macrophage-free isolated system was utilized. MC3T3-E1 cells were seeded into 6-well plates. Upon reaching 80% confluence, the medium was replaced with OM. The experiment was strictly divided into 3 groups: Ctrl (OM only), LEV-CM (OM mixed with intact LEV-conditioned medium), and OM+rmIL-10 (OM directly supplemented with rmIL-10). The administration concentration of rmIL-10 was strictly matched to the physiological level previously quantified in the intact LEV-CM. After mineralization induction, total RNA and proteins were extracted for reverse transcription quantitative polymerase chain reaction (RT-qPCR) and Western blot analysis.

### Cell immunofluorescence

To evaluate the expression and localization of specific proteins within cells, immunofluorescence staining was performed in this study. Cells were seeded and cultured on slides or in culture dishes. They were then washed with PBS and fixed with 4% PFA for 15 min at room temperature. For intracellular proteins, the fixed cells were thoroughly washed with PBS and permeabilized with 0.1% Triton X-100 solution for 10 min at room temperature. For membrane-bound proteins, the permeabilization step was omitted. To minimize nonspecific binding, the cells were blocked with a solution containing 5% normal goat serum for 1 h at room temperature. The diluted specific primary antibodies (e.g., anti-protein A antibody) were incubated overnight at 4 °C. On the following day, after washing with PBS, the cells were incubated with the corresponding fluorescently labeled secondary antibodies for 1 h at room temperature in the dark.

For LEV internalization studies, LEVs were first prelabeled with the fluorescent dye 3,3′-dioctadecyloxacarbocyanine perchlorate (DiO; no. MB4239, MeilunBio) according to the manufacturer’s instructions. These DiO-labeled LEVs were then coincubated with cells for a specified duration of 6 h. After incubation, cells were washed rigorously with PBS to remove unbound LEVs and then fixed with 4% PFA. The cell membranes were stained with the fluorescent dye 1,1′-dioctadecyl-3,3,3′,3′-tetramethylindocarbocyanine perchlorate to observe whether the LEVs had entered the cells.

The cell nuclei were counterstained with DAPI for 5 min, and the slides were mounted with an antifade mounting medium. All sections were observed and imaged using a Leica STELLARIS fluorescence microscope.

### Flow cytometry analysis

Mouse gingival tissue samples from the buccal and palatal sides of the second molar were collected under sterile conditions and minced into 1-mm^3^ fragments. The tissue fragments were then placed in a digestion solution containing collagenase and dispase and incubated in a 37 °C water bath with gentle shaking until complete tissue dissociation. The resulting cell suspension was filtered through a 40-μm cell strainer to remove any undigested tissue fragments. To remove red blood cells, the cell pellet was subsequently treated with red blood cell lysis buffer. For surface marker detection, the prepared single-cell suspension was resuspended in flow cytometry buffer containing 2% FBS and labeled with phycoerythrin (PE)-conjugated anti-F4/80 antibody (Elabscience, no. E-AB-F0995D) and PE/C7-conjugated anti-CD163 antibody (BD Biosciences, no. 155319) for 30 min at 4 °C in the dark. After washes, data acquisition was performed using a flow cytometer (Cytomics FC 500, Beckman Coulter), and the percentage and phenotype of different cell subsets were analyzed using the FlowJo software (v10.8.1).

For the flow cytometry analysis of RAW264.7 cell phenotypes, the cells were first collected from the culture plates by gentle scraping and then washed 3 times with pre-chilled PBS to obtain a single-cell suspension. The cells were then resuspended in flow cytometry buffer containing 2% FBS. The cells were subsequently incubated with antibodies, including allophycocyanin-conjugated anti-CD86 antibody (Elabscience, no. E-AB-F0994E), allophycocyanin-conjugated anti-CD206 antibody (Elabscience, no. E-AB-F1135E), and PE/C7-conjugated anti-CD163 antibody (BD Biosciences, no. 155319), for 30 min at 4 °C in the dark. To ensure proper staining of CD206, which can be detected as an intracellular protein, the permeabilization step was performed using the Fix & Perm Kit (Liankebio, China). After washing with flow cytometry buffer, data were acquired on a flow cytometer. All data were analyzed using the FlowJo software.

### Cell Counting Kit-8 assay for cell viability

RAW264.7 cells were seeded at an appropriate density into 96-well culture plates. After the cells had fully adhered, the original medium was removed, and fresh medium containing 200 ng/ml *Pg*LPS was added to establish an inflammatory model. Subsequently, the cells were treated with different concentrations of LEVs (5, 10, 15, 20, and 30 μg/ml). After 24 h, the culture medium was aspirated, and the cells were gently washed 3 times with PBS. Finally, 100 μl of serum-free high-glucose DMEM and Cell Counting Kit-8 (CCK-8) solution were added to each well. After a 1-h incubation at 37 °C, the absorbance was measured at 450 nm using a microplate reader.

### FAO assay

The FAO Assay Kit (Assay Genie, no. BR00001) was used to quantify the level of cellular FAO. Briefly, RAW264.7 cells were seeded into 96-well culture plates. Following the application of various treatments, the FAO reaction was initiated and terminated in strict accordance with the manufacturer’s instructions. Subsequently, the absorbance at 492 nm was measured on a microplate reader to reflect the cellular FAO level.

### Real-time PCR

Total RNA was extracted using TRIzol reagent (Invitrogen). Complementary DNA was synthesized using a reverse transcription kit (Takara). The reactions were performed on an RT-qPCR instrument (LightCycler 480, Roche) with SYBR Green qPCR Mix (Vazyme, no. Q311-02). The primer sequences are detailed in Table S1. Relative gene expression was calculated using the 2^−ΔΔCt^ method, with β-actin serving as an internal reference.

### Protein extraction and Western blot

Total protein or nuclear protein was extracted depending on the experimental objective. Total protein was extracted using the radioimmunoprecipitation assay (RIPA) lysis method. For nuclear protein, the Nuclear Protein Extraction Kit (no. P0027; Beyotime, China) was used. Tissue protein extraction was performed as follows: gingival tissues were carefully isolated from the maxillary region near the second molars of mice. The tissues were immediately placed in pre-chilled RIPA lysis buffer and homogenized using a sterile pestle on ice. The homogenized lysates were then centrifuged at 12,000 rpm for 15 min at 4 °C to pellet cellular debris. The resulting supernatant, containing the total protein, was carefully collected for subsequent analysis or stored at −80 °C.

Following extraction, protein concentrations were determined using a bicinchoninic acid protein assay kit (P0009, Beyotime, China). Western blot analysis was performed to detect protein expression in cells or tissues. Equal amounts of protein samples (20 to 30 μg) were separated by sodium dodecyl sulfate–polyacrylamide gel electrophoresis (SDS-PAGE) and then transferred to a polyvinylidene difluoride membrane. The membrane was blocked with 5% nonfat milk for 1 h at room temperature. Subsequently, the membrane was incubated with a corresponding primary antibody at 4 °C overnight. On the following day, the membrane was washed 3 times with tris-buffered saline with Tween 20 for 5 min each and then incubated with a horseradish peroxidase-labeled secondary antibody for 1 h at room temperature. Finally, protein bands were visualized using an enhanced chemiluminescence substrate, and densitometric analysis of the bands was performed using the ImageJ software.

### Enzyme-linked immunosorbent assay

Cell culture supernatants were collected and analyzed by enzyme-linked immunosorbent assay (ELISA) to assess cytokine levels. Specifically, the levels of mouse IL-10 (no. RK00016, ABclonal) and IL-6 (no. RK00008, ABclonal) were measured using commercially available ELISA kits according to the manufacturer’s instructions.

### Chromatin immunoprecipitation assay

Chromatin immunoprecipitation (ChIP) was performed using the Pierce Magnetic ChIP Kit (Thermo Scientific, no. 26157) to detect the binding of the target transcription factor to the promoter region of the gene of interest. Briefly, cells were cross-linked with 1% formaldehyde, and the reaction was quenched with glycine. After cell lysis and micrococcal nuclease digestion, the chromatin was sonicated to fragments of approximately 200 to 1,000 bp. The fragmented chromatin was incubated with the primary antibody at 4 °C overnight, with an equal amount of immunoglobulin G serving as a negative control. Protein A/G Magnetic Beads were then added to precipitate the antigen–antibody–DNA complex. After washing, the complex was eluted and de-cross-linked. Finally, the DNA was extracted for subsequent qPCR analysis. The xenobiotic response element (XRE) primers used were as follows: 5′-TGAGTCGGCAAAATTTGAGC-3′ and antisense 5′-CATAATCAGCCTGTGTAGAT-3′.

### Overexpression and knockdown of genes

To construct the overexpression vectors, the coding sequences of Cpt1a were first amplified from cell complementary DNA by PCR. The Cpt1a PCR product was digested with 2 restriction enzymes and then ligated into a 3×FLAG-tagged expression vector. The ligation product was transformed into *Escherichia coli* DH5α, and single clones were picked for plasmid extraction. The correct recombinant plasmids were finally selected for transfection experiments. The verified recombinant plasmid was cotransfected with lentiviral packaging plasmids (e.g., psPAX2 and pMD2.G) into HEK293T cells for the efficient production and collection of lentiviral particles. The collected lentiviral supernatant was concentrated and titrated and then used to infect (transduce) the target cells, macrophages. The lentiviral system thus achieved stable and efficient overexpression of Cpt1a in macrophages, which were subsequently used for functional experiments. In addition, we designed and constructed specific small interfering RNA (siRNA) sequences targeting CPT1A and NQO1, with the detailed sequences listed in Table S2.

For transfection, cells were seeded at an appropriate density into 6-well plates or other suitable culture plates based on experimental requirements. When cell confluence reached 30%, the original medium was removed, and the overexpression or siRNA plasmids were transfected into the cells using Lipofectamine 3000 (Invitrogen). After 72 h of transfection, the transfection efficiency was verified by Western blot.

### Coimmunoprecipitation

To investigate the interaction between a target protein and its potential interacting partners, as well as its ubiquitination level, a coimmunoprecipitation (Co-IP) experiment was performed. First, cell or tissue samples were collected, and total protein was extracted using RIPA lysis buffer (supplemented with protease and phosphatase inhibitors). Equal amounts of total protein lysate were coincubated with a primary antibody against the target protein or an isotype immunoglobulin G control at 4 °C overnight. Subsequently, Protein A/G Magnetic Beads were added and incubated at 4 °C overnight to capture the antigen–antibody complexes. The beads were then collected using a magnetic rack and washed 3 to 5 times with a cold wash buffer (containing 1% NP-40) to remove nonspecifically bound proteins. Finally, the beads were resuspended in SDS-PAGE loading buffer and heated at 95 °C for 5 to 10 min to denature the proteins, followed by Western blot analysis. In the Western blot, the target protein was verified using its specific primary antibody, while the interaction was detected using a primary antibody against the potential interacting partner. For ubiquitination detection, an anti-ubiquitin antibody was used to identify the ubiquitinated form of the target protein.

### Tryptophan-targeted metabolomics

To evaluate the effect of LEVs on the intracellular tryptophan metabolic pathway in macrophages, targeted metabolomic analysis was performed. First, RAW264.7 cells were subjected to different treatments. After treatment, the culture medium was removed, and the cells were washed with pre-chilled PBS. Intracellular metabolites were then extracted by lysing the cells with pre-chilled 80% methanol/water solution (containing an internal standard). The lysate was sonicated and centrifuged, and the supernatant was collected and dried under a stream of nitrogen. The samples were then redissolved in mobile phase and analyzed using a liquid chromatography–tandem mass spectrometry (LC–MS/MS) system. The mass spectrometer was operated in positive ion mode with multiple reaction monitoring for the quantitative analysis of target metabolites. All data were qualitatively and quantitatively analyzed by comparison with standards and normalized using the internal standard. Finally, statistical analysis was performed to compare the relative levels of metabolites among different treatment groups.

### Bioinformatics analysis

This study analyzed the GSE16134 dataset from the National Center for Biotechnology Information Gene Expression Omnibus (NCBI GEO) database, which includes 310 gingival tissue samples from patients who underwent periodontal surgery. After obtaining the gene expression matrix, we used single-sample gene set enrichment analysis (ssGSEA) [24] to calculate gene set scores for glycolysis, FAO, inflammatory levels, and the macrophage M1/M2 ratio for each sample. These scores were used to quantify the biological state within different periodontitis tissue samples.

To investigate the potential mechanism of LEVs on macrophages, we analyzed the GSE274800 dataset, which contains RNA sequencing (RNA-seq) data from BMDMs treated with BEVs or PBS. We performed differential gene expression analysis using the DESeq2 R package [25], comparing the LEV-treated group to the control group. Subsequently, we used the clusterProfiler R package [26] for functional enrichment analysis of the differentially expressed genes (DEGs). Furthermore, to evaluate the effect of LEVs on the tryptophan metabolic pathway, we used the gsva R package [27] to calculate the scores of tryptophan metabolism-related gene sets.

To investigate the function of AhR in macrophages, we performed differential gene expression analysis on the GSE221093 dataset. This dataset contains RNA-seq data from AhR knockout mouse BMDMs and wild-type BMDMs, with and without LPS stimulation. We used the DESeq2 R package to compare cells under different genotypes and treatment conditions to identify gene expression changes related to AhR-mediated lipid mediator production. In addition, we performed differential gene expression analysis on the GSE181249 dataset, which includes transcriptomic data from human peripheral blood mononuclear cells transfected with AhR-specific siRNA or control siRNA. We used the DESeq2 R package to analyze these transcriptomic data to identify gene expression changes induced by AhR silencing.

This study also analyzed the assay for transposase-accessible chromatin using sequencing (ATAC-seq) data from the GSE221144 dataset, which provides information on chromatin accessibility in AhR knockout and wild-type macrophages. We plotted the ATAC-seq track for the NQO1 gene region to visualize its chromatin accessibility status in different genotypes. Additionally, we retrieved the Arnt:Ahr binding motif (TBGCACGCAA) from the Homer database and used the scanMotifGenomeWide.pl program (version 4.11) from Homer to perform a genome-wide scan in the mouse mm10 reference genome to predict potential AhR binding sites.

### Statistical analysis

Data are presented as mean ± standard deviation. Statistical analysis was performed using the GraphPad Prism software (version 10.1). The Student *t* test was used for comparisons between 2 groups. One-way analysis of variance followed by Tukey’s post hoc test was used for comparisons among multiple groups. Differences were considered statistically significant when the *P* value is <0.05.

## Results

### Isolation and characterization of LEVs and their effects on macrophage

**Fig. 1.**
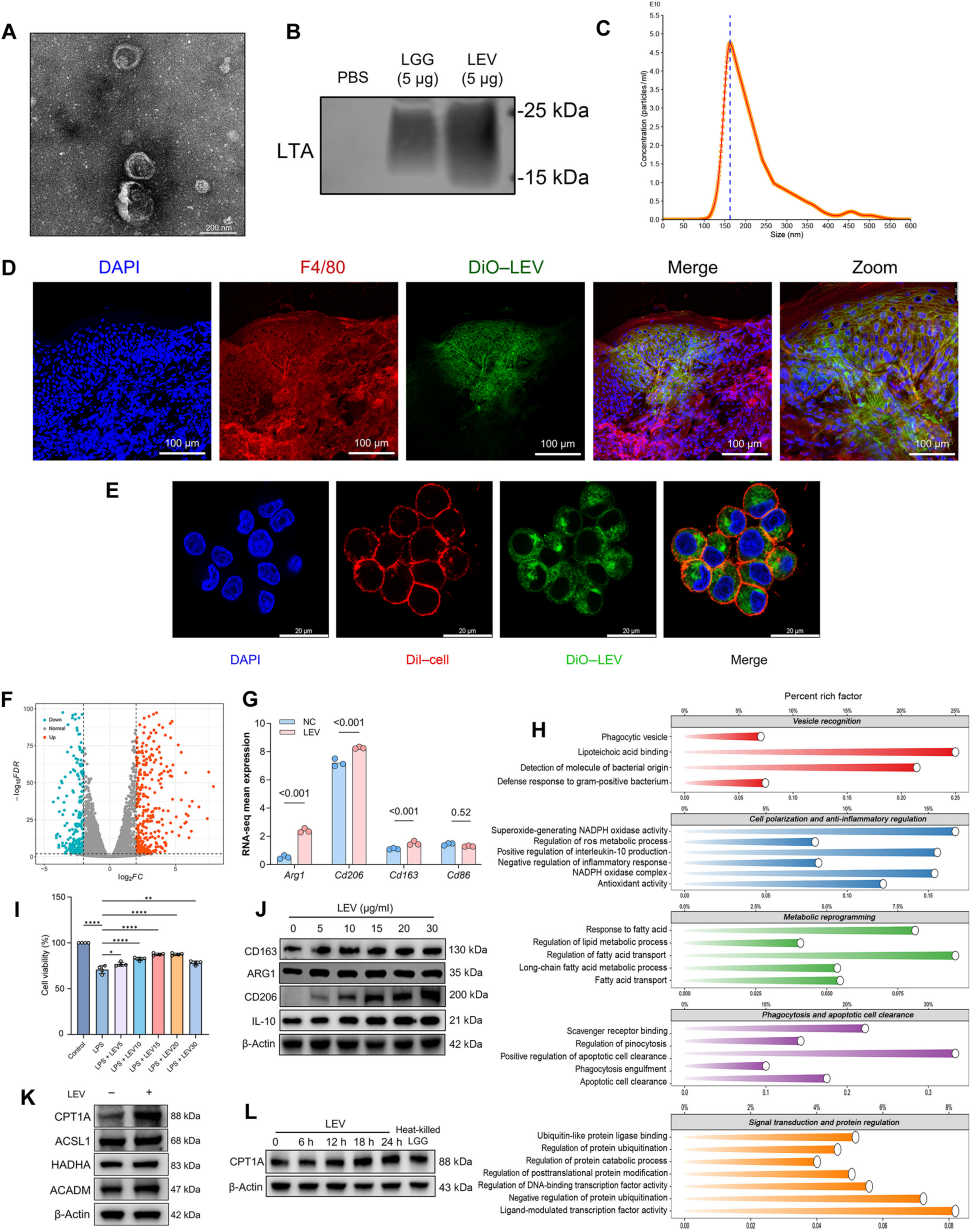
Isolation, characterization, cellular uptake of *Lactobacillus rhamnosus* GG-derived extracellular vesicles (LEVs) and phenotypic analysis of LEV-treated macrophages. (A) Transmission electron microscopy (TEM) image of isolated LEVs, showing their shapes with a double-layered membrane structure. (B) Western blot analysis showing the bacterial origin of LEVs. An anti-lipoteichoic acid (anti-LTA) antibody was used to detect the presence of LTA in LEVs and their parental bacteria *L. rhamnosus* GG (*LGG*) lysate, with the phosphate-buffered saline (PBS) group serving as a negative control. (C) Nanoparticle tracking analysis (NTA) graph showing the particle size distribution of isolated LEVs. (D) Immunofluorescence images showing the uptake of 3,3′-dioctadecyloxacarbocyanine perchlorate (DiO)-labeled LEVs (green) by gingival macrophages (red, F4/80^+^) 24 h after injection into the periodontal tissue of a mouse model. Nuclei were stained with 4′,6-diamidino-2-phenylindole (DAPI; blue). (E) In vitro immunofluorescence images showing the uptake of DiO-labeled LEVs (green) by macrophages (membrane stained red with 1,1′-dioctadecyl-3,3,3′,3′-tetramethylindocarbocyanine perchlorate [DiI]). Nuclei were stained with DAPI (blue). (F) Volcano plot showing differentially expressed genes (DEGs) in LEV-treated macrophages compared to control cells. (G) Bar plot showing the expression levels of M1 and M2 macrophage signature genes in LEV-treated cells. (H) Gene Ontology enrichment analysis of the DEGs, revealing enrichment in terms related to vesicle recognition, cell polarization and anti-inflammatory regulation, metabolic reprogramming, phagocytosis and apoptotic cell clearance, signal transduction, and protein regulation. (I) Cell Counting Kit-8 (CCK-8) assay results showing the effect of different concentrations of LEVs on the viability of macrophages challenged with lipopolysaccharide (LPS). (J) Western blot analysis showing the protein expression levels of M2 polarization markers (arginase-1 [ARG1], CD206, CD163, and interleukin-10 [IL-10]) in macrophages treated with different concentrations of LEVs. (K) Western blot analysis of the expression levels of key fatty acid oxidation (FAO) enzymes in LEV-treated macrophages. (L) Western blot analysis demonstrating the time-dependent effect of LEVs on carnitine palmitoyltransferase 1A (CPT1A) protein expression in macrophages. Additionally, a control group treated with heat-inactivated *LGG* (56 °C for 30 min) is included. *P < 0.05, **P < 0.01, and ****P < 0.0001 in the indicated groups.

The isolated LEVs exhibited typical nanoscale, spherical, or irregular vesicular shapes with a clearly discernible double-layered membrane structure and uniform morphology (Fig. 1A). Western blot analysis identified the presence of LTA in both *LGG* lysates and purified LEVs, which was entirely absent in PBS and PM controls (Fig. 1B and Fig. S1A). NTA further revealed the particle size distribution of the LEVs, showing a primary peak diameter at 162.5 nm (Fig. 1C). Furthermore, a detergent lysis assay was performed to evaluate the structural integrity of the lipid bilayer. Following treatment with 0.1% Triton X-100, NTA indicated a reduction in nanoparticle concentration from 4.8 × 10^10^ to 5.0 × 10^9^ particles/ml. This detergent sensitivity indicates that the isolated particles are predominantly enveloped by intact lipid bilayers (Fig. S1B).

To deeply investigate the cellular uptake of LEVs both in vivo and in vitro, we used the DiO green dye to label the LEVs. In the periodontitis mouse model, DiO-labeled LEVs were observed to have penetrated the periodontal tissue, with LEVs localized within gingival macrophages (Fig. 1D). Immunofluorescence results further confirmed that the labeled LEVs were effectively taken up by the macrophages in vitro, with their green fluorescence signal clearly present in the cytoplasm inside the cell membrane (Fig. 1E). These results collectively confirm that LEVs can be internalized by a key immune cell, namely, the macrophage.

We further analyzed gene expression profiling on LEV-treated macrophages. The DEG analysis results showed that a total of 425 genes were up-regulated and 241 genes were down-regulated (Fig. 1F). LEVs were found to significantly up-regulate the expression of multiple M2-type macrophage marker genes, including *Arg1*, *Cd206*, and *Cd163*, while the expression of the M1 polarization marker *Cd86* showed no significant difference, suggesting their ability to induce macrophages to polarize toward a pro-reparative phenotype (Fig. 1G). Gene Ontology enrichment analysis revealed that these differential genes were primarily enriched in several important biological function terms, including vesicle recognition, signal transduction and protein regulation, cell polarization and anti-inflammatory regulation, phagocytosis and clearance of apoptotic cells, and metabolic reprogramming (Fig. 1H).

The effect of LEVs on the viability of *Pg*LPS (subsequently referred to as LPS) induced macrophages was detected by the CCK-8 method (Fig. 1I). Compared to the control group, treatment with LPS alone significantly reduced cell viability. However, LEVs dose-dependently rescued the LPS-induced decline in macrophage viability, peaking at a concentration of 15 μg/ml. Additionally, the protein expression levels of M2 polarization markers, including ARG1, CD206, CD163, and IL-10, were detected in macrophages treated with different concentrations of LEVs. Compared to those of the control group, the protein expression levels of these markers were all up-regulated in the LEV-treated group (Fig. 1J).

Based on the hint of metabolic reprogramming in the Gene Ontology enrichment analysis, we focused on the FAO pathway and detected the expression of key FAO enzymes: CPT1A, ACSL1, HADHA, and ACADM. These proteins were all up-regulated to varying degrees after LEV treatment, with the most substantial increase in the expression of CPT1A (Fig. 1K). CPT1A expression peaked at 18 h post-LEV treatment (Fig. 1L). Conversely, inactivated *LGG* failed to induce this CPT1A up-regulation, indicating that LEV-mediated FAO regulation strictly depends on inherent vesicle bioactivity.

### Periodontitis tissues exhibit a metabolic shift toward glycolysis and away from FAO

**Fig. 2.**
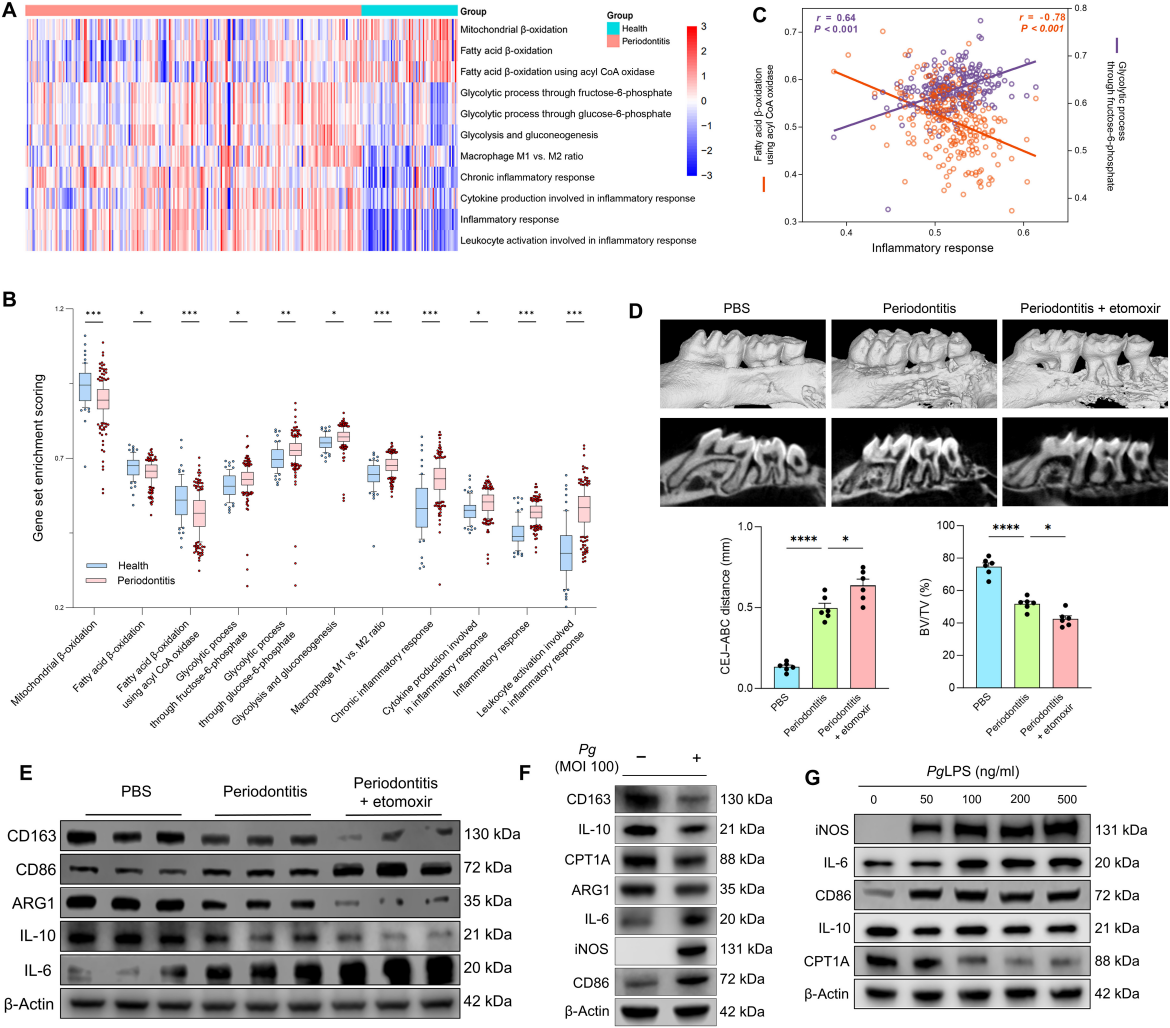
Metabolic profiling and comparative analysis of glycolysis and fatty acid oxidation (FAO) in periodontitis tissues. (A) Single-sample gene set enrichment analysis (ssGSEA) method scores of metabolic pathways and immune-related signatures in periodontitis and healthy tissue samples. The heatmap shows the relative enrichment of glycolysis, inflammatory response, M1/M2 macrophage ratio, and FAO pathways. (B) Boxplots showing the statistical difference in the enrichment scores for each pathway between the healthy control and periodontitis groups. (C) Scatter plots with bivariate linear regression analysis showing the correlation between inflammatory response and FAO (left) as well as between inflammatory response and glycolysis (right). The correlation coefficient (*r*) and *P* value for each correlation are indicated. (D) Representative reconstructed and 3-dimensional (3D) scanned sections along the longitudinal direction of the maxilla. The distance (mm) between the cementoenamel junction (CEJ) and the alveolar bone crest (ABC) and the percentage of bone volume/total volume (BV/TV) were analyzed. BV/TV represents the bone volume fraction, that is, bone volume over tissue volume. (E) Western blot analysis of macrophage polarization markers (CD163, CD86, arginase-1 [ARG1], interleukin-10 [IL-10], and interleukin-6 [IL-6]) in periodontitis tissue, with or without etomoxir treatment, and in the phosphate-buffered saline (PBS) control group. (F) The expression of CD163, IL-10, carnitine palmitoyltransferase 1A (CPT1A), ARG1, IL-6, inducible nitric oxide synthase (iNOS), and CD86 in RAW264.7 cells stimulated with *Porphyromonas gingivalis* (multiplicity of infection [MOI] 100) were detected by Western blot analysis. (G) The expressions of iNOS, IL-6, CD86, IL-10, and CPT1A in RAW264.7 cells stimulated with increasing concentrations of *Pg*LPS (50, 100, 200, and 500 ng/ml) were detected by Western blot analysis. *P < 0.05, **P < 0.01, ***P < 0.001, and ****P < 0.0001 in the indicated groups.

To investigate the metabolic status within the periodontitis microenvironment, we analyzed the gene expression profiles from bulk RNA-seq of 310 periodontal tissue samples. ssGSEA showed that the activity of glycolysis, inflammatory response, and the M1/M2-ratio-related pathways were all significantly up-regulated in the periodontitis tissues (Fig. 2A and B). In addition, the FAO pathway was significantly down-regulated in the periodontitis group. Figure 2C reveals a significant negative correlation between the inflammatory response and FAO (correlation coefficient *r* = −0.78) and a significant positive correlation with glycolysis (correlation coefficient *r* = 0.64). These bioinformatics analyses indicate that the periodontitis tissue microenvironment exhibits enhanced inflammation, up-regulated glycolysis, and inhibited FAO.

To further validate the role of FAO in periodontitis, we established a periodontitis mouse model. The micro-CT analysis revealed that the etomoxir (inhibitor of FAO) treated periodontitis group exhibited exacerbated bone resorption compared to the periodontitis-only group (Fig. 2D). Etomoxir treatment further increased the CEJ–ABC distance (from 0.49 ± 0.07 to 0.63 ± 0.09 mm; PBS: 0.13 ± 0.02 mm) and decreased the BV/TV ratio (from 51.85% ± 3.92% to 42.55% ± 4.75%; PBS: 74.81% ± 5.56%). In addition, Western blot showed a decrease in M2 markers (CD163, ARG1, and IL-10) and an increase in M1 polarization marker (CD86) and inflammatory marker (IL-6). In the etomoxir-treated periodontitis group, the expression levels of CD163, ARG1, and IL-10 were decreased compared to those in the periodontitis-only group, while the expression of CD86 and IL-6 were up-regulated (Fig. 2E and Fig. S1C).

Next, we employed the major periodontal pathogen *Pg* as a straightforward in vitro technique to replicate the inflammatory microenvironment. *Pg* stimulation led to a decrease in macrophage M2 markers (CD163, IL-10, ARG1) and CPT1A, while the expression of M1 markers (IL-6, iNOS, and CD86) was up-regulated (Fig. 2F). Similarly, LPS treatment dose-dependently increased iNOS, IL-6, and CD86 expression, with a concomitant dose-dependent decrease in IL-10 and CPT1A levels (Fig. 2G).

### LEVs promote macrophage M2 polarization and bone regeneration by enhancing CPT1A-mediated FAO

**Fig. 3.**
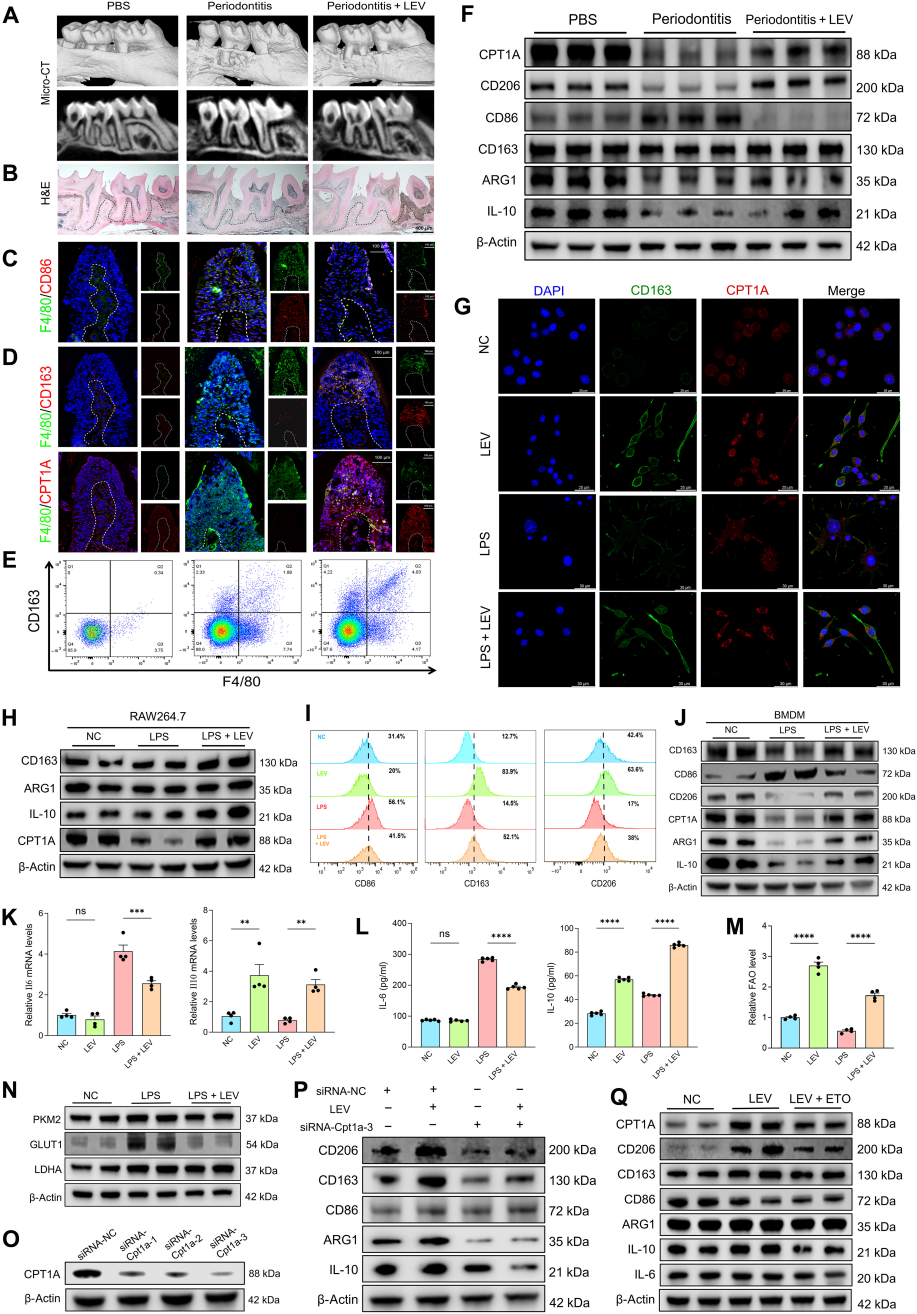
Evaluation of macrophage polarization, alveolar bone, and carnitine palmitoyltransferase 1A (CPT1A)-mediated fatty acid oxidation (FAO) following *Lactobacillus rhamnosus* GG-derived extracellular vesicle (LEV) treatment. (A) Representative micro-computed tomography (micro-CT) images of the maxillary alveolar bone, evaluating bone loss in the periodontitis and LEV-treated groups. (B) Histological analysis of periodontal tissue sections with hematoxylin and eosin (H&E) staining. (C) Immunofluorescence images of periodontal tissue, showing macrophage infiltration and the labeling of CD86^+^ M1 macrophages. (D) Immunofluorescence images showing the labeling of CD163^+^ M2 macrophages and the expression of CPT1A in periodontal tissue. (E) Representative flow cytometric plots were used to evaluate the proportion of CD163^+^ M2 macrophage (F4/80^+^) subset in periodontal tissues. (F) The expressions of CPT1A, CD206, CD86, CD163, arginase-1 (ARG1), and interleukin-10 (IL-10) in the gingiva of mice were detected by Western blot analysis. (G to I) RAW264.7 cells were exposed to lipopolysaccharide (LPS), with or without LEV treatment. (G) Representative immunofluorescence images showing the morphology of macrophages and the expression of CD163 and CPT1A after different treatments. (H) Western blot analysis of macrophage polarization markers (CD163, ARG1, IL-10, and CPT1A) in RAW264.7 cells after LPS and LEV cotreatment. (I) Flow cytometry analysis to assess the proportions of different macrophage subsets (CD86^+^, CD163^+^, and CD206^+^) after LEV treatment. (J) Western blot analysis showing the protein expression levels of M1 and M2 markers in primary bone-marrow-derived macrophage (BMDM) cells. (K) Bar plots showing the messenger RNA (mRNA) expression levels of interleukin-6 (IL-6) and IL-10 in LPS-stimulated macrophages after LEV treatment. (L) Enzyme-linked immunosorbent assay (ELISA) results showing the secretion levels of IL-6 and IL-10 from macrophages under different treatment conditions. (M) Bar plot showing the effect of LEV treatment on FAO activity in macrophages under control and LPS-stimulated conditions. (N) Western blot analysis of glycolysis-related proteins (pyruvate kinase M2 [PKM2], glucose transporter 1 [GLUT1], and lactate dehydrogenase A [LDHA]) in macrophages. (O) Western blot analysis showing the knockdown efficiency of CPT1A using 3 different small interfering RNAs (siRNAs). (P) Western blot analysis showing the effect of CPT1A knockdown on the expression of macrophage polarization proteins after LEV treatment. (Q) Western blot analysis demonstrating the effect of the FAO inhibitor etomoxir on LEV-induced macrophage polarization protein expression. **P < 0.01, ***P < 0.001, and ****P < 0.0001 in the indicated groups.

We investigated the in vivo therapeutic effect of LEVs on periodontitis. Micro-CT imaging showed that the periodontitis group exhibited significant alveolar bone destruction, characterized by an increased CEJ–ABC distance and a decrease in the BV/TV ratio (Fig. 3A and Fig. S1D). In contrast, LEV treatment effectively rescued this pathological condition, leading to a reduction in bone loss. H&E staining showed severe inflammatory cell infiltration in the periodontitis group compared to the PBS group, while LEV treatment diminished the inflammatory infiltrate (Fig. 3B). Immunofluorescence analysis indicated that LEVs reduced the overall macrophage count and attenuated CD86^+^ M1 macrophage infiltration while increasing the presence of CD163^+^ M2 macrophages and CPT1A expression within the periodontal tissue (Fig. 3C and D). Flow cytometry analysis further substantiated the effect of LEVs on macrophage polarization. Compared to the periodontitis group, the proportion of the CD163^+^ M2 macrophage subpopulation was significantly up-regulated in the LEV-treated group (Fig. 3E and Fig. S1E). Consistently, Western blot analysis demonstrated that LEVs reversed the periodontitis-induced down-regulation of M2 markers (CPT1A, CD163, ARG1, IL-10, and CD206) and the up-regulation of M1 marker (CD86) (Fig. 3F and Fig. S1F).

In vitro, LEVs induced a morphological transition from the LPS-stimulated round M1-like phenotype to an elongated spindle M2-like phenotype, with a concomitant increase in CD163 and CPT1A expression (Fig. 3G). In RAW264.7 cells (Fig. 3H and Fig. S1G), LPS treatment significantly down-regulated the protein levels of CD163, ARG1, IL-10, and CPT1A. However, cotreatment with LEVs and LPS significantly up-regulated the expression of these M2 markers and CPT1A. A similar trend was observed in primary BMDMs (Fig. 3J and Fig. S1H), where LEVs significantly up-regulated M2 marker expression and down-regulated M1 marker expression. Flow cytometry verified that LEVs reversed the LPS-promoted M1 polarization, showing a reduced proportion of CD86^+^ cells and increased proportions of CD163^+^ and CD206^+^ cells (Fig. 3I and Fig. S1I). Finally, the effect of LEVs on inflammatory cytokine production by macrophages was evaluated. Figure 3K shows that LEVs could reduce the messenger RNA (mRNA) expression of the pro-inflammatory cytokine IL-6 and promote the mRNA expression of the anti-inflammatory cytokine IL-10 in LPS-stimulated macrophages. LEVs attenuated LPS-induced IL-6 production and promoted IL-10 secretion while having no effect on basal IL-6 levels in unstimulated macrophages (Fig. 3L).

Metabolically, LEVs increased FAO activity under both control and inflammatory conditions (Fig. 3M) and down-regulated glycolysis-related proteins (pyruvate kinase M2, glucose transporter 1, and lactate dehydrogenase A) (Fig. 3N and Fig. S2A). To define the mechanistic role of FAO, CPT1A was knocked down via siRNA (Fig. 3O). The CPT1A knockdown or pharmacological inhibition of FAO by etomoxir significantly abrogated the ability of LEVs to promote M2 polarization and inhibit M1 markers (Fig. 3P and Q and Fig. S2B). These results indicate that LEV-mediated macrophage reprogramming is dependent on the activation of the FAO pathway.

### LEVs promote osteogenesis through IL-10 released from M2 macrophages

**Fig. 4.**
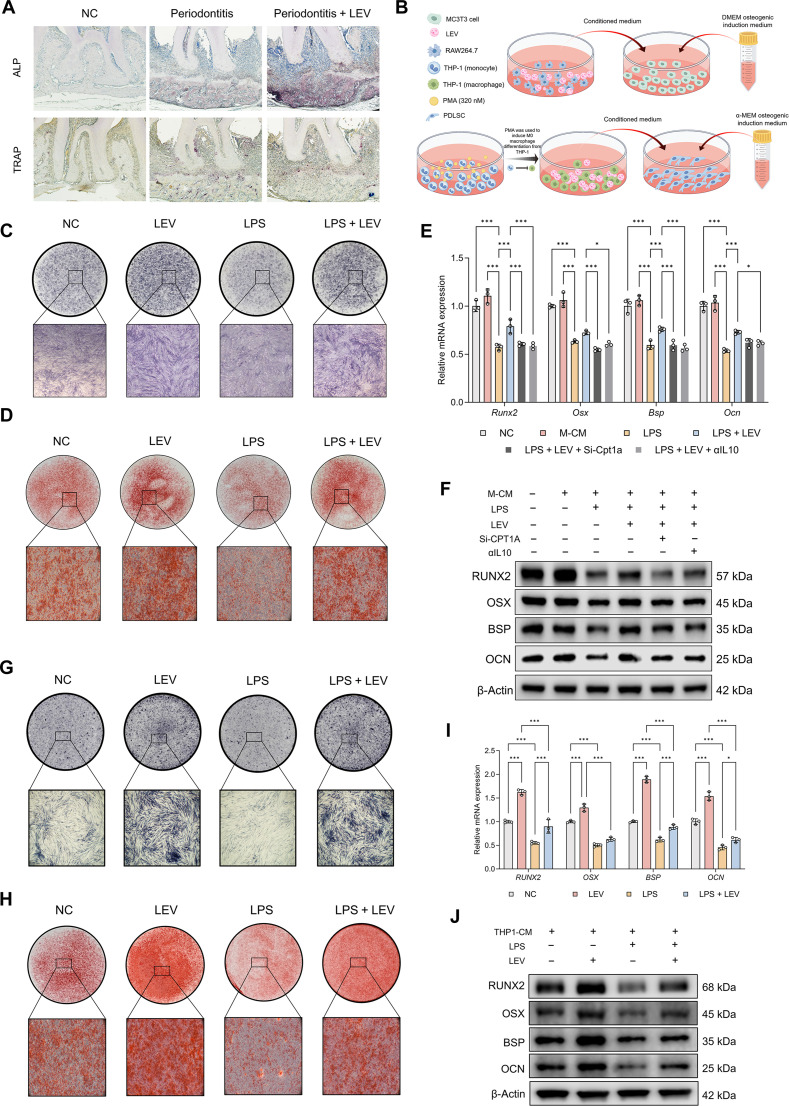
Assessment of interleukin-10 (IL-10) secretion from macrophages and in vitro osteogenic responses upon *Lactobacillus rhamnosus* GG-derived extracellular vesicle (LEV) treatment. (A) Histochemical staining for alkaline phosphatase (ALP; osteoblast marker) and tartrate-resistant acid phosphatase (TRAP; osteoclast marker) on paraffin sections of mouse maxillae from the periodontitis model. (B) A schematic diagram illustrating the in vitro co-culture systems (RAW264.7/MC3T3-E1 and THP-1/periodontal ligament stem cells [PDLSCs]) used to evaluate the paracrine pro-osteogenic effect of LEVs via macrophages. (C) ALP staining of MC3T3-E1 cells cultured in conditioned media (CM) derived from RAW264.7 macrophages treated with different conditions (NC, lipopolysaccharide [LPS], LEV, and LPS + LEV). (D) Alizarin Red S (ARS) staining of MC3T3-E1 cells cultured in CM derived from RAW264.7 macrophages treated with different conditions (NC, LPS, LEV, and LPS + LEV) to evaluate mineralized nodule formation. (E) Quantitative polymerase chain reaction (qPCR) analysis to detect the gene expression levels of osteogenic markers (*Runx2*, *Osx*, *Bsp*, and *Ocn*) in MC3T3-E1 cells cultured in CM derived from RAW264.7 macrophages treated with different conditions (NC, LPS, LEV, LPS + LEV, siCPT1A, and αIL10). (F) Western blot analysis to detect the protein expression levels of osteogenic markers in MC3T3-E1 cells cultured in CM derived from RAW264.7 macrophages treated with different conditions (NC, LPS, LEV, LPS + LEV, siCPT1A, and αIL10). (G) ALP staining of PDLSCs cultured in CM derived from THP-1 macrophages treated with different conditions (NC, LPS, LEV, and LPS + LEV). (H) ARS staining of PDLSCs cultured in CM derived from THP-1 macrophages treated with different conditions (NC, LPS, LEV, and LPS + LEV) to evaluate mineralized nodule formation. (I) qPCR analysis to detect the gene expression levels of osteogenic markers (*RUNX2*, *OSX*, *BSP*, and *OCN*) in PDLSCs cultured in CM derived from THP-1 macrophages treated with different conditions (NC, LPS, LEV, and LPS + LEV). (J) Western blot analysis to detect the protein expression levels of osteogenic markers in PDLSCs cultured in CM derived from THP-1 macrophages treated with different conditions (NC, LPS, LEV, and LPS + LEV). *P < 0.05 and ***P < 0.001 in the indicated groups.

To investigate the role of LEVs in osteogenesis, we evaluated bone metabolism markers in paraffin sections of mouse maxillae. ALP and TRAP staining indicated that osteoclast activity was elevated in the periodontitis group, reflecting an imbalance between alveolar bone resorption and formation. Conversely, LEV treatment attenuated osteoclast differentiation while simultaneously promoting osteoblast-mediated bone repair (Fig. 4A). To further characterize this regenerative process, Masson’s trichrome staining and immunofluorescence analysis were performed. The results indicated enhanced collagen matrix deposition and a localized up-regulation in the expression of osteogenic markers, RUNX2 and OCN, in the LEV-treated group compared to that in the periodontitis group (Fig. S2C). These static histological data, combined with the micro-CT structural parameters, indicate that LEVs facilitate bone repair by modulating periodontal bone metabolism.

To further elucidate the role of macrophages in LEV-induced osteogenesis in vitro, RAW264.7/MC3T3-E1 and THP-1/PDLSC co-culture systems were used (Fig. 4B). In the RAW264.7–MC3T3-E1 system, ALP and ARS staining results demonstrated that compared to the LPS-treated group CM, the CM from LEV-treated macrophages enhanced the ALP activity and mineralization capacity of MC3T3-E1 cells, indicating that the macrophage supernatant stimulated by LEVs possesses a potent pro-osteogenic effect (Fig. 4C and D). In addition, qPCR and Western blot analyses were performed to examine the expression of key osteogenic markers (RUNX2, OSX, BSP, and OCN) in MC3T3-E1 cells. The results showed that the CM from the LEV-treated macrophage group up-regulated the expression of these osteogenic genes and proteins compared to that in the LPS-treated group (Fig. 4E and F).

A previous study indicated an association between CPT1A and IL-10 expression [28]. To investigate the specific molecular mechanism by which LEVs promotes osteogenesis, we additionally included 2 experimental groups: one in which CPT1A was knocked down in macrophages with siRNA prior to LEV stimulation and another in which a neutralizing anti-IL-10 antibody was added to the CM from LEV-treated macrophages. The results revealed that in both of these groups, the LEV-induced up-regulation of osteogenic gene expression was significantly inhibited. This suggests that LEVs indirectly promote osteoblast differentiation through CPT1A-dependent metabolic reprogramming and the subsequent paracrine secretion of IL-10. To evaluate whether the osteogenic effect is mediated directly by IL-10 or requires other factors in the CM, an isolated osteoblast culture system was utilized. Osteoblasts were treated with standard OM (Ctrl), LEV-CM, or OM supplemented with rmIL-10 (OM+rmIL-10). The qPCR and Western blot data indicate that rmIL-10 alone increased the mRNA and protein expression levels of early osteogenic markers compared to the Ctrl group. However, the specific up-regulation induced by rmIL-10 alone was lower than that induced by the complete LEV-CM (Fig. S2D and E). These findings indicate that IL-10 exerts a direct osteogenic effect on osteoblasts, while the participation of other secretory factors within the LEV-CM is required to mediate the overall osteogenic capacity.

Subsequently, we validated these findings using the THP-1 and PDLSC co-culture system. Similar to the RAW264.7/MC3T3-E1 co-culture method, we treated THP-1 cells with LPS or LEVs and cultured PDLSCs in CM from macrophage. ALP and ARS results showed that compared to that from the LPS-treated macrophage group, the CM from the LEV-treated macrophage group also promoted the ALP activity and mineralization capacity of PDLSCs (Fig. 4G and H). The qPCR and Western blot results for osteogenic markers were consistent with our earlier findings, confirming that the macrophage supernatant from the LEV-treated group can promote the osteogenic differentiation of PDLSCs (Fig. 4I and J).

### LEVs inhibit the 26S proteasome-mediated degradation of CPT1A

**Fig. 5.**
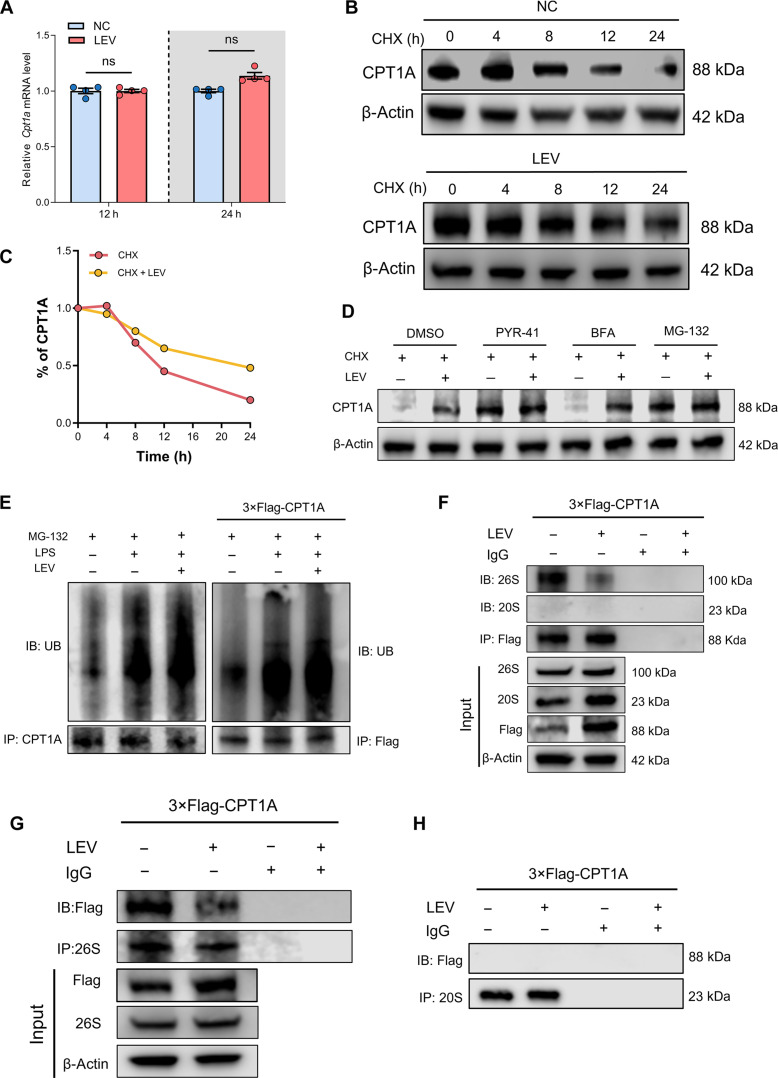
Analysis of carnitine palmitoyltransferase 1A (CPT1A) protein stability and 26S proteasome-mediated degradation in the presence of *Lactobacillus rhamnosus* GG-derived extracellular vesicles (LEVs). (A) Quantitative polymerase chain reaction (qPCR) analysis to detect *CPT1A* messenger RNA (mRNA) expression levels in macrophages 12 and 24 h after LEV treatment. (B and C) Cycloheximide (CHX) chase assays to evaluate the effect of LEV treatment on the half-life of CPT1A protein in macrophages, with time points at 0, 4, 8, 12, and 24 h. (D) CHX chase assays with different degradation pathway inhibitors (lysosome inhibitor bafilomycin A1 [BFA], proteasome inhibitor MG-132, and E1 ubiquitin-activating enzyme inhibitor PYR-41) to investigate the specific pathway by which LEVs enhance CPT1A stability. (E) Coimmunoprecipitation (Co-IP) analysis to detect the ubiquitination levels of endogenous or exogenously overexpressed 3×Flag-CPT1A after LEV treatment. (F) Co-IP analysis using a Flag antibody for immunoprecipitation to detect the physical interaction between 3×Flag-CPT1A and the 26S or 20S proteasome. (G) Reverse Co-IP analysis using a 26S proteasome antibody for immunoprecipitation to validate the interaction between CPT1A and the 26S proteasome. (H) Co-IP analysis using a 20S proteasome antibody for immunoprecipitation, serving as a negative control. ns indicates no statistical significance.

To investigate the mechanism by which LEVs up-regulates CPT1A protein expression in macrophages, we first determined CPT1A mRNA expression by qPCR after LEV treatment. The results showed no significant changes in CPT1A transcriptional level compared to the control group (Fig. 5A), suggesting that LEV-mediated regulation of CPT1A may occur at the posttranslational level. We further employed a CHX chase assay to evaluate the effect of LEVs on the half-life of CPT1A protein (Fig. 5B and C). Western blot analysis demonstrated that CPT1A protein degradation was attenuated in the LEV-treated group compared to that in the control group, and its protein half-life was markedly prolonged. Furthermore, the lysosome inhibitor BFA failed to prevent CPT1A degradation alone, whereas cotreatment with LEVs increased CPT1A levels (Fig. 5D). In contrast, both the proteasome inhibitor MG132 and the E1 ubiquitin-activating enzyme inhibitor PYR-41 effectively stabilized CPT1A protein on their own, with no additive effect when cotreated with LEVs. These results indicate that LEVs enhance CPT1A stability by inhibiting the ubiquitin–proteasome pathway, rather than the autophagy–lysosome pathway.

To investigate whether LEVs influence CPT1A through its ubiquitination level, we performed Co-IP assays to directly detect CPT1A ubiquitination (Fig. 5E). The results revealed that LPS treatment enhanced the ubiquitination level of CPT1A; however, subsequent LEV treatment did not lead to a further increase in its ubiquitination level. These findings suggest that LEV-mediated regulation of CPT1A may not depend on ubiquitination modification, but rather on a direct action upon the proteasome itself. To determine CPT1A physically interacts with the proteasome, we conducted Co-IP assays in cells stably overexpressing 3×Flag-CPT1A. The results showed that 3×Flag-CPT1A coimmunoprecipitated with the 26S proteasome but not the 20S proteasome, confirming a physical association between CPT1A and the 26S proteasome (Fig. 5F). Furthermore, we observed that the amount of coimmunoprecipitated 26S proteasome was decreased in the LEV-treated group compared to that in the control, suggesting that LEVs might interfere with the interaction between CPT1A and the 26S proteasome. To corroborate this finding, we performed a reverse Co-IP using a 26S proteasome antibody for immunoprecipitation (Fig. 5G). As a negative control, immunoprecipitation with 20S proteasome antibody failed to coimmunoprecipitate CPT1A (Fig. 5H).

### LEVs activate the l-tryptophan–kynurenine–AhR axis to regulate CPT1A expression

**Fig. 6.**
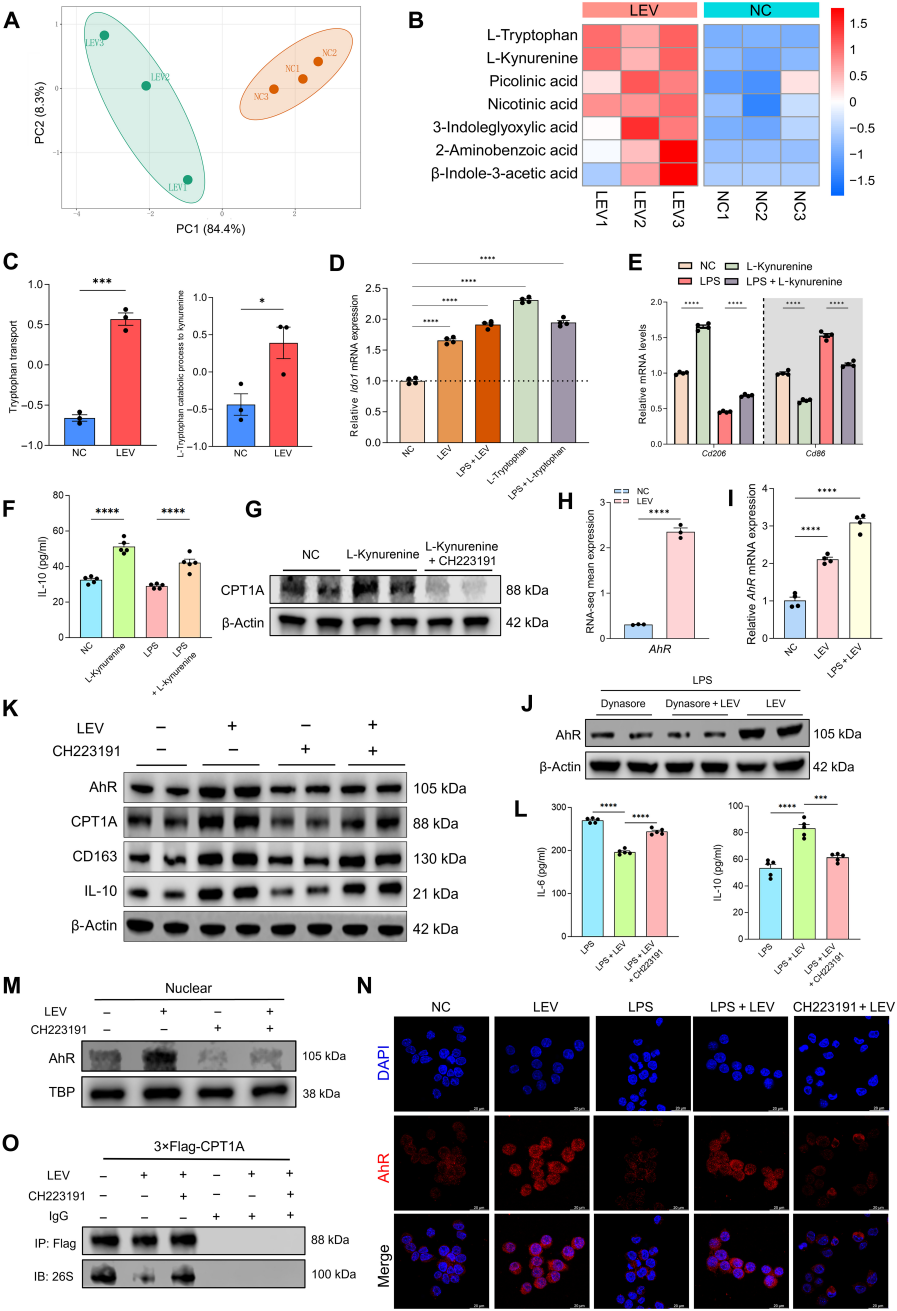
Measurement of the l-tryptophan–kynurenine–aryl hydrocarbon receptor (AhR) axis and carnitine palmitoyltransferase 1A (CPT1A) expression levels under *Lactobacillus rhamnosus* GG-derived extracellular vesicle (LEV) treatment. (A) Principal component analysis plot from tryptophan-targeted metabolomic analysis, showing the metabolic profile differences between LEV-treated macrophages and the control group. (B) A heatmap showing the levels of l-tryptophan and its downstream metabolite l-kynurenine with statistical in LEV-treated macrophages. (C) Single-sample gene set enrichment analysis (ssGSEA) analysis plot showing the gene set enrichment of the l-tryptophan catabolic process to the kynurenine pathway and the tryptophan transport pathway in LEV-treated macrophages. (D) Quantitative polymerase chain reaction (qPCR) analysis to detect the messenger RNA (mRNA) expression level of the key tryptophan metabolic enzyme indoleamine 2,3-dioxygenase 1 (IDO1) in macrophages treated with LEVs and l-tryptophan, with or without lipopolysaccharide (LPS)-induced inflammation. (E) qPCR analysis to detect the mRNA expression of M1 (CD86) and M2 (CD206) markers in macrophages treated with l-kynurenine. (F) Enzyme-linked immunosorbent assay (ELISA) analysis to detect the protein levels of IL-10 in macrophages treated with l-kynurenine, with or without LPS-induced inflammation. (G) Western blot analysis to detect the effect of the AhR-specific inhibitor CH223191 on l-kynurenine-induced CPT1A protein expression in macrophages. (H) RNA sequencing (RNA-seq) analysis showing the gene expression level of AhR in LEV-treated macrophages. (I) qPCR analysis to detect the mRNA expression levels of AhR in macrophages treated with LEV and LPS + LEV. (J) Western blot analysis to evaluate the effect of the vesicular endocytosis inhibitor Dynasore on LEV-induced intracellular AhR expression in the LPS inflammatory environment. (K) Western blot analysis to detect the effect of the AhR inhibitor CH223191 on LEV-induced CPT1A, CD163, and interleukin-10 (IL-10) protein expression. (L) ELISA analysis to detect the effect of the AhR inhibitor CH223191 on the secretion levels of interleukin-6 (IL-6) and IL-10 in LEV-treated macrophages. (M) Western blot analysis to detect the protein expression levels of nuclear AhR in LEV-treated macrophages. (N) Immunofluorescence images showing the subcellular localization of AhR in macrophages after treatment with the control, LPS, LEV, LPS + LEV, and CH223191 + LEV groups. (O) Coimmunoprecipitation (Co-IP) analysis to detect the effect of the AHR inhibitor CH223191 on the interaction between CPT1A and the 26S proteasome. *P < 0.05, ***P < 0.001, and ****P < 0.0001 in the indicated groups.

Previous metabolomic study based on LC–MS showed that tryptophan and its derivatives are abundant in the more than 2,000 metabolic components of LEVs [21]. Thus, to investigate the upstream regulatory mechanism by which LEVs up-regulates CPT1A expression in macrophages, we performed tryptophan-targeted metabolomic analysis on macrophages treated with LEVs. The principal component analysis showed a difference in the metabolic profile between the LEV-treated group and the control group (Fig. 6A). The levels of both l-tryptophan and its downstream metabolite l-kynurenine were increased in macrophages treated with LEVs (Fig. 6B). We further performed ssGSEA scoring on RNA-seq data for related pathways. The results showed that LEV treatment activated the l-tryptophan catabolic process to kynurenine and tryptophan transport pathways (Fig. 6C).

To further confirm the effect of LEVs on l-tryptophan metabolism, we measured the mRNA expression level of the key tryptophan metabolic enzyme *Ido1*. The results showed that LEV treatments up-regulated *Ido1* mRNA expression (Fig. 6D). In addition, *Cd86* expression was lower in the l-kynurenine groups than in the control groups, while *Cd206* expression was higher. This indicates that l-kynurenine could promote the M2 polarization of macrophages, which is consistent with the anti-inflammatory effect of IL-10 (Fig. 6E). ELISA results also showed that both l-kynurenine and LPS + l-kynurenine up-regulated IL-10 protein levels (Fig. 6F). Western blot analysis further showed that l-kynurenine treatment up-regulated CPT1A protein expression, and this effect was inhibited by the addition of the AhR-specific inhibitor CH223191, suggesting a key role for AhR (Fig. 6G).

We then verified the effect of LEVs on AhR expression. RNA-seq analysis showed that the gene expression level of AhR was significantly up-regulated after LEV treatment of macrophages (Fig. 6H). Next, qPCR results showed that LEV treatment significantly up-regulated both the mRNA expression levels of *AhR* (Fig. 6I). A previous study showed that inflammatory stimuli such as LPS can also induce intracellular AhR expression [29]. To exclude LPS as a confounding factor, we further investigated whether LEVs directly activate AhR through endocytosis. For this purpose, in the inflammatory environment, we pre-treated macrophages with Dynasore, a validated small-molecule inhibitor of vesicular endocytosis, to effectively inhibit LEV uptake [30]. Our results revealed that upon the inhibition of LEV endocytosis by Dynasore, the expression level of intracellular AhR decreased (Fig. 6J).

To determine the AhR dependency of LEV effects, RAW264.7 cells and primary BMDMs were utilized. In RAW264.7 cells, LEV-induced up-regulation of CPT1A, CD163, and IL-10 was attenuated by the AhR inhibitor CH223191 (Fig. 6K). The cell-intrinsic role of AhR was further verified in primary BMDMs via Ahr-siRNA (Fig. S2F). Ahr knockdown abolished the LEV-induced stabilization of CPT1A and inhibited the expression of CD163 and IL-10. The ELISA results further confirmed that in the LPS + LEV-treated group, the pro-inflammatory factor IL-6 was decreased while the anti-inflammatory factor IL-10 was increased (Fig. 6L). After adding CH223191, IL-6 levels returned to baseline and IL-10 levels decreased. We also assessed the effect of LEVs on AhR subcellular localization through Western blot and immunofluorescence. The results showed that LEV treatment increased AhR protein levels in the nucleus (Fig. 6M), and immunofluorescence also showed that compared to that in the control and LPS groups, AhR was translocated to the nucleus in the LEVs and LPS + LEV groups (Fig. 6N). When CH223191 was added, AhR nuclear localization was inhibited, and the protein was mainly distributed in the perinuclear region. The activation of AhR by LEVs suggests its potential role as a transcription factor that enters the nucleus to regulate the expression of downstream genes.

To elucidate the effect of AhR activation on CPT1A stability, we investigated the association of CPT1A with the proteasome by Co-IP using a Flag antibody to detect 26S proteasome subunits (Fig. 6O). The results showed that compared to the LEV-only treatment group, the amount of 26S proteasome coimmunoprecipitated with CPT1A increased after adding the AhR inhibitor CH223191, suggesting that inhibition of AhR may lead to enhanced CPT1A degradation.

### LEVs activate the AhR/NQO1 axis to maintain CPT1A protein stability in macrophages

**Fig. 7.**
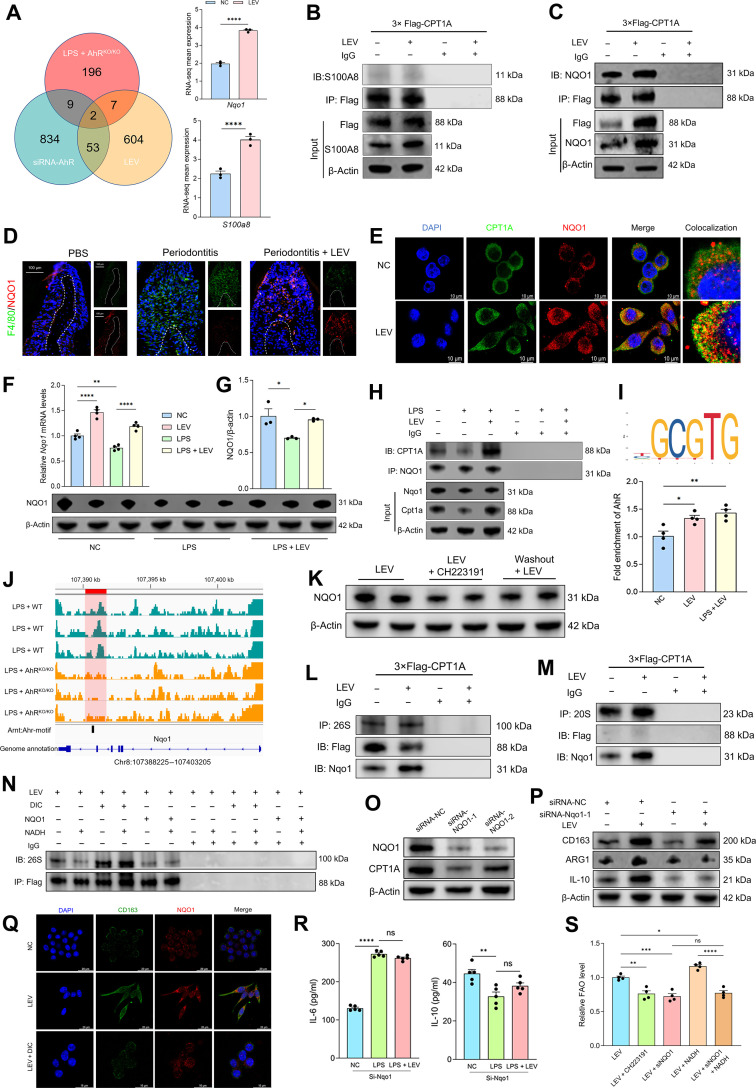
Investigation of the aryl hydrocarbon receptor (AhR)/NAD(P)H:quinone oxidoreductase 1 (NQO1) signaling pathway and carnitine palmitoyltransferase 1A (CPT1A) protein stability in *Lactobacillus rhamnosus* GG-derived extracellular vesicle (LEV)-treated macrophages. (A) Venn diagram showing the intersection of differentially expressed genes (DEGs) from 3 datasets: macrophages from lipopolysaccharide (LPS)-treated AhR knockout mice, small interfering RNA (siRNA)-AhR-treated macrophages, and LEV-treated macrophages, used for screening downstream target genes of AhR. (B and C) Coimmunoprecipitation (Co-IP) analysis to detect the physical interaction between 3×Flag-CPT1A and S100A8 or NQO1. (D) Immunofluorescence staining of NQO1 and F4/80 in periodontal tissue, used to evaluate the effect of LEV intervention on NQO1 expression in macrophages. (E) Cellular immunofluorescence staining to observe the colocalization of NQO1 and CPT1A in macrophages after LEV stimulation. (F) Quantitative polymerase chain reaction (qPCR) analysis to detect the effect of LEVs on NQO1 messenger RNA (mRNA) expression levels in macrophages with or without LPS-induced inflammation. (G) Western blot analysis to detect the effect of LEVs on NQO1 protein expression levels in macrophages with or without LPS-induced inflammation. (H) Co-IP analysis to investigate the effect of LEVs on the interaction between NQO1 and CPT1A in an LPS inflammatory environment. (I) Chromatin immunoprecipitation (ChIP)–qPCR analysis to detect the effect of LEVs on the recruitment of AhR to the NQO1 promoter. (J) Assay for transposase-accessible chromatin using sequencing (ATAC-seq) traces of the *Nqo1* gene locus in wild-type and unstimulated or LPS-stimulated bone-marrow-derived macrophages (BMDMs). The AhR/Arnt binding motif (xenobiotic response element [XRE]) in this region of the mouse genome is shown in the lower trace. (K) Washout experiment demonstrating the rescue effect of the AhR inhibitor CH223191 on LEV-mediated regulation of NQO1 protein expression. (L) Co-IP analysis to evaluate the effect of LEV treatment on the formation of a complex between CPT1A, NQO1, and the 26S proteasome. (M) Co-IP analysis to evaluate the binding of NQO1 and CPT1A to the 20S proteasome, serving as a negative control. (N) Co-IP analysis to investigate the role of NQO1 and its coenzyme nicotinamide adenine dinucleotide + hydrogen (NADH; 100 μM for 4 h) in regulating the interaction between CPT1A and the 26S proteasome. Cells were also treated with dicumarol (400 μM) for 4 h. (O) Western blot analysis to evaluate the knockdown efficiency of 2 NQO1 siRNAs. (P) Western blot analysis to evaluate the effect of NQO1 knockdown on the expression of M2 polarization-related proteins (CD163, arginase-1 [ARG1], and interleukin-10 [IL-10]) in macrophages. (Q) Cellular immunofluorescence staining to observe the effect of the NQO1 inhibitor dicumarol on LEV-induced M2 polarization and NQO1 expression. (R) Enzyme-linked immunosorbent assay (ELISA) analysis to evaluate the effect of NQO1 knockdown on LEVs’ regulation of interleukin-6 (IL-6) and IL-10 secretion in macrophages in an inflammatory environment. (S) Fatty acid oxidation (FAO) activity analysis to investigate the roles of AhR and NQO1 in LEV-induced macrophage metabolic reprogramming, showing the effects of 5 treatment groups (LEV, LEV + CH223191, LEV + siNQO1, LEV + NADH, and LEV + siNQO1 + NADH). *P < 0.05, **P < 0.01, ***P < 0.001, and ****P < 0.0001 in the indicated groups.

We then investigated the mechanism by which LEV-mediated AhR activation affects CPT1A protein stability. We first used bioinformatics to screen for AhR downstream target genes from 3 independent bulk RNA-seq datasets. By intersecting the DEGs from AhR knockout mice, siRNA-AhR-treated, and LEV-treated macrophages, we identified 2 potential downstream targets: S100A8 and NQO1. In the RNA-seq data of LEV-treated macrophages, both genes were significantly up-regulated in the LEV group (Fig. 7A). To determine which gene physically interacts with CPT1A, we performed Co-IP experiments. The results showed that only NQO1 could coprecipitate with 3×Flag-CPT1A (Fig. 7B and C), suggesting that NQO1, rather than S100A8, is the key mediator of AhR’s regulation of CPT1A. We also performed tissue immunofluorescence staining in the periodontitis mouse model, finding that NQO1 expression was reduced in macrophages (F4/80^+^) in the periodontitis group but was enhanced after LEV intervention (Fig. 7D). Cellular immunofluorescence experiments further confirmed that the colocalization fluorescence signal of NQO1 and CPT1A was stronger in LEV-treated macrophages than in the control group (Fig. 7E).

To further investigate the regulatory effect of LEVs on NQO1, we conducted experiments in an in vitro inflammatory environment. qPCR and Western blot results showed that LEVs could still significantly up-regulate NQO1 mRNA and protein expression levels under LPS stimulation (Fig. 7F and G). More importantly, the binding of NQO1 to CPT1A was reduced in the inflammatory environment, but LEV treatment could effectively restore this binding (Fig. 7H). To confirm the direct regulatory relationship of AhR on NQO1, we performed a ChIP–qPCR experiment. The results showed that LEVs promoted the recruitment of AhR to a specific XRE site on the NQO1 promoter, regardless of LPS stimulation (Fig. 7I), confirming that LEVs increase NQO1 transcription by activating AhR. Additionally, ATAC-seq data also indicated that in an inflammatory environment, AhR knockout in macrophages reduced the accessibility of the NQO1 gene (Fig. 7J). We also demonstrated that the regulatory effect of LEVs on NQO1 is reversible through a washout experiment with the AhR inhibitor CH223191. Specifically, when CH223191 was washed out and LEVs were reintroduced, NQO1 protein levels were restored (Fig. 7K).

To clarify how NQO1 regulates CPT1A stability, we studied their complex formation with the 26S proteasome. Co-IP results showed that the 26S proteasome could form a complex with both CPT1A and NQO1. After LEV treatment, CPT1A bound to the 26S proteasome decreased, while NQO1 bound to the 26S proteasome increased (Fig. 7L). As a negative control, the 20S proteasome bound only to NQO1 and failed to bind to CPT1A (Fig. 7M). This suggests that NQO1 may prevent CPT1A degradation by forming a tripartite complex with CPT1A and the 26S proteasome. We further confirmed through Co-IP that NQO1 relies on its coenzyme NADH to modulate the binding of CPT1A to the 26S proteasome. The results showed that the addition of NADH reduced the binding of CPT1A to the 26S proteasome, while the NQO1 inhibitor dicumarol enhanced this binding. In the presence of dicumarol, the addition of NADH could not reduce the binding of CPT1A to the 26S proteasome. These results collectively suggest that NADH may assist NQO1 in maintaining CPT1A stability (Fig. 7N).

Finally, we verified the critical role of NQO1 in macrophage function. We used siRNA to knock down NQO1 (Fig. 7O) and found that the ability of macrophages to polarize to the M2 type (CD163, ARG1, and IL-10) was inhibited (Fig. 7P and Fig. S2G). Furthermore, cellular immunofluorescence experiments showed that LEV-induced M2 polarization and NQO1 expression could be inhibited by the NQO1 inhibitor dicumarol (Fig. 7Q). In an inflammatory environment, NQO1 knockdown caused LEVs to lose their anti-inflammatory effect, manifested by increased IL-6 levels and decreased IL-10 levels (Fig. 7R). Functional experiments further demonstrated that LEVs promote macrophage FAO levels through the AhR and NQO1 pathway. Both the AhR inhibitor CH223191 and NQO1 knockdown significantly reduced the LEV-induced FAO levels, while the addition of NADH enhanced FAO (Fig. 7S).

## Discussion

An increasing number of studies have shown that the health benefits of probiotics are largely mediated by the extracellular vesicles (EVs) that they produce [31–33]. As a subtype of BEVs, LEVs offer new insights into tissue regeneration and the treatment of inflammatory diseases. This study delved into the molecular mechanisms of LEVs in the treatment of periodontitis and indicated that LEVs regulate macrophage polarization and function through the tryptophan–kynurenine–AhR signaling axis. Furthermore, we also found the mechanism by which LEVs maintains the stable level of a key FAO protein, CPT1A. This regulation not only promotes the enhancement of FAO levels in macrophages, thereby facilitating their polarization from pro-inflammatory (M1) to pro-reparative (M2) phenotypes, but also ultimately mediates osteogenic repair of periodontal tissue. Collectively, our findings provide a molecular basis for the anti-inflammatory and pro-reparative effects of LEVs as a novel postbiotic and also offer new insights into the therapeutic potential of metabolic reprogramming for bone regeneration in inflammatory environments.

Probiotics have emerged as promising adjunct therapies for periodontitis; their widespread application is hindered by significant inherent challenges [34,35]. For instance, the survival and effective colonization of live bacteria in the complex oral microenvironment are challenging. The therapeutic effects of even the same probiotic species can vary significantly due to strain-specific characteristics and individual differences, which largely restricts their clinical predictability and reliability. As a postbiotic, BEVs are progressively overcoming these challenges, providing a safer, more efficient, and more clinically translatable strategy. Regarding delivery feasibility, compared to live probiotics, BEVs, as cell-free nanoparticles, have a lipid bilayer membrane structure that effectively protects their internal bioactive molecules, enabling them to remain highly stable in harsh environments [36,37]. LEVs retain the bioactive constituents of their parental bacteria while possessing nanoscale dimensions and a robust lipid bilayer, enabling superior penetration through the gingival epithelial barrier compared to whole bacteria. EVs are not inhibited by antibiotics, making them ideal for combined use to clear pathogens while synergistically exerting their potent anti-inflammatory and immunomodulatory effects [37]. Regarding safety, unlike live probiotics, which carry inherent physical risks of opportunistic bacteremia and uncontrolled colonization in immunocompromised patients, LEVs are non-replicating, ensuring a highly controllable safety profile. Furthermore, when compared to mammalian-cell-derived EVs (e.g., mesenchymal stem cell EVs), LEVs exhibit significant translational advantages, including highly scalable bacterial fermentation and substantially lower manufacturing costs.

In the pathological process of chronic inflammatory diseases, the phenotypic transition of macrophages from inflammatory initiators to reparative executors is tightly regulated by cellular metabolic reprogramming, particularly FAO. Our previous studies have also confirmed that enhancing FAO levels can promote cementum mineralization and thus accelerate cementum regeneration [38]. Our results showed that etomoxir treatment led to more severe bone destruction and a stronger inflammatory response, which confirmed the crucial role of FAO in the resolution of periodontitis inflammation. Importantly, our research found that LEVs can effectively drive macrophage metabolic reprogramming, further polarizing them into a reparative M2 phenotype. Mechanistically, we found that upon entering macrophages, LEVs up-regulate intracellular l-kynurenine. As a degradation product of tryptophan metabolism, l-kynurenine’s up-regulation was further confirmed in our study by the activation of IDO1. l-Kynurenine induces a conformational change in AhR, transitioning it into an active state, ultimately allowing it to be recognized by nuclear transport proteins and enter the nucleus to exert its transcriptional regulatory function. Recent studies have confirmed that AhR plays a cell-intrinsic role in the development, differentiation, survival, and maintenance of various immune cell types, highlighting the pivotal function of this molecule in regulating physiological homeostasis and immune responses [39–41]. Specifically in the oral cavity, deleting AhR from macrophages exacerbates ligation-induced periodontitis, whereas AhR activation provides robust protection against alveolar bone loss [42]. While our results demonstrate the role of the l-kynurenine–AhR signaling axis, it is imperative to objectively acknowledge the inherent molecular heterogeneity of LEV cargoes. LEVs encapsulate a vast array of microbe-derived molecules; therefore, it is highly probable that alternative classical signaling networks, such as short-chain fatty acid receptor activation and Toll-like receptor cascades, are simultaneously engaged during LEV–macrophage interactions. We do not exclude the parallel activation of these alternative pathways, which may synergistically contribute to the ultimate osteogenic rescue. Nevertheless, the substantial abrogation of LEV-induced macrophage reprogramming and osteogenesis following specific AhR blockade (via AhR-siRNA and CH223191) demonstrates that the AhR signaling node serves as a dominant and indispensable rate-limiting pathway within this highly complex multireceptor network. Future multi-omics investigations are warranted to delineate the intricate synergistic cross talk between the primary AhR axis and alternative LEV-driven signaling cascades.

Consistent with the MISEV2023 guidelines, we recognize that LEVs are highly heterogeneous; our study strategically prioritized the regulation of metabolites. The specific reasons are as follows: First, previous studies have demonstrated that *LGG* mediates its immunomodulatory effects primarily by producing high-affinity AhR ligands. Gu et al. [21] identified that LEVs contain over 2,000 metabolites, with tryptophan and its derivatives being abundantly present in the LEV cargo. Therefore, we hypothesized that LEVs function as specialized concentrated reservoirs and delivery vectors for these microbial metabolic signatures. Second, while many research studies currently focus on proteins and nucleic acid molecules within EVs [43], metabolites are components of EV cargo, and less attention has been paid to metabolites. Third, a key advantage of metabolites is their ability to be directly absorbed by host cells and activate receptors, thereby bypassing complex cellular mechanisms. More importantly, our observation that AhR blockade largely abolishes the therapeutic efficacy of LEVs supports the conclusion that these encapsulated metabolites function as the primary signaling drivers in this context. Although current physical fractionation techniques render the absolute isolation of metabolites from coencapsulated proteins or RNAs technically unfeasible, future integrative multi-omics investigations are warranted to elucidate potential synergistic effects of the LEV proteome or sRNAs; however, the present data suggest a mechanistic association between EV-delivered metabolites and macrophage plasticity.

To elucidate the molecular mechanism underlying the LEV-induced up-regulation of CPT1A, we investigated its posttranslational modification. We observed that LEVs inhibited CPT1A degradation without altering the overall polyubiquitination status (Fig. 5E). Mechanistically, previous studies have indicated that NQO1 can directly bind to target proteins, such as p53 [44] and c-Fos [45], acting as a spatial gatekeeper in an NADH-dependent manner to protect them from proteasomal degradation. Concurrently, both our experimental results and the existing literature [46] demonstrate that NQO1 directly interacts with the 26S proteasome. Furthermore, evidence shows that the 26S proteasome can directly execute protein degradation independent of ubiquitin tags [47,48]. Our data indicate that LEVs enhance the physical interaction between NQO1 and the 26S proteasome subunit, and this interaction is regulated by NADH (Fig. 7N). Synthesizing these mechanisms, we hypothesize that NQO1 functions as a structural gatekeeper on the 26S proteasome. By providing steric hindrance, NQO1 competitively prevents CPT1A from entering the catalytic chamber, thereby inhibiting its ubiquitin-independent degradation despite the unchanged ubiquitination levels.

It is essential to acknowledge several limitations in this study. First, the dosage of LEVs was standardized by total protein concentration in our study, which is currently the mainstream and most reproducible metric in EV research. While tryptophan metabolites were identified as the key bioactive payload in this study, we acknowledge that protein-based dosing may not precisely quantify the specific metabolite loading. This represents a limitation that warrants further exploration of multidimensional quantification strategies in future investigation. Second, although this study demonstrated the critical role of the AhR signaling pathway in LEV-mediated macrophage metabolic reprogramming and alveolar bone protection through in vitro siRNA knockdown and in vivo administration of the specific antagonist CH223191, the current in vivo experimental model possesses inherent methodological limitations. Future investigations urgently require the introduction of macrophage-specific AhR conditional knockout murine models (e.g., Lyz2-Cre;Ahr fl/fl) to precisely dissect the absolute proportional contribution of LEVs in delaying alveolar bone resorption via host macrophage-intrinsic mechanisms within the complex in vivo pathological environment. Third, because the primary objective of this study was biological functional validation rather than the development of a drug delivery system, naked LEVs were utilized for periodontal tissue microinjections. Currently, there is no unified consensus on the standardized dosage and administration frequency for EV-based local therapies. Therefore, the specific local injection regimen (50 μg of LEVs every 3 d) was strictly formulated by referencing established preclinical models [20,49]. However, because this protocol serves strictly as a preclinical pharmacological strategy, the evaluation of the precise pharmacokinetics and dynamic biodistribution of naked LEVs within this highly vascularized tissue remains an unaddressed limitation. To bridge the gap between biological proof of concept and true clinical translation, future investigations must strictly transition to advanced drug delivery systems, incorporating LEVs into functionalized, tissue-adhesive biomaterials to anchor the cargo, reduce administration frequency, and enable rigorous future pharmacokinetic tracking. Fourth, in evaluating the stability of CPT1A, our CHX chase assays indicated a prolonged half-life following LEV treatment. While our Co-IP data (Fig. 7L and N) revealed altered complex formation between CPT1A and the 26S proteasome, direct quantitative assessments of cell-free 26S proteasome activity are lacking in the present study, which represents a methodological limitation.

## Ethical Approval

All animal procedures were performed with the approval of the Ethics Committee of The Hospital of Stomatology, Wuhan University (S07925060D).

## Data Availability

Sequencing data are mostly obtained from the NCBI GEO database (https://www.ncbi.nlm.nih.gov/geo/). All other data supporting the findings of this study are available from the corresponding author upon reasonable request.

## Supplementary Materials

Supplementary 1 Graphical Abstract Figs. S1 and S2 Tables S1 and S2
