# Supplementary material for: Tryptophan-Enriched Lactobacillus rhamnosus GG-derived Nanovesicles Promote Alveolar Bone Regeneration through Macrophage Fatty Acid Oxidation
Source: Biomater Res. 2026 Jul 17;30:0370. doi: 10.34133/bmr.0370 (PMC13376381; doi:10.34133/bmr.0370)
Supplement: Supplementary 1 — Graphical Abstract Figs. S1 and S2 Tables S1 and S2 [file bmr.0370.f1.zip › Supplementary Table S1-2.docx]

| **Table S1** | |  |
| --- | --- | --- |
|  | | |
| **Name** | **F/R** | **Sequences** |
| *Il6* | F | AGCCAGAGTCCTTCAGAGAGAT |
|  | R | AGGAGAGCATTGGAAATTGGGG |
| *Il10* | F | AGGCGCTGTCATCGATTTCTC |
|  | R | GCCTTGTAGACACCTTGGTCTTG |
| *Cpt1a* | F | TGTGGACCTGCATTCCTTCC |
|  | R | CCAGCACAAAGTTGCAGGAC |
| *Ido1* | F | ATGTGGGCTTTGCTCTACCA |
|  | R | AGCTGCCCGTTCTCAATCAG |
| *Cd206* | F | GTGGACGCTCTAAGTGCCAT |
|  | R | GAATCTGACACCCAGCGGAA |
| *Cd86* | F | ATGGACCCCAGATGCACCAT |
|  | R | CGGCAGATATGCAGTCCCAT |
| *AhR* | F | CTGGCAGGATTTGCAAGAAGG |
|  | R | ATTCCGCCCGGTCTTCTGTA |
| *Nqo1* | F | CATTGCAGTGGTTTGGGGTG |
|  | R | TCTGGAAAGGACCGTTGTCG |
| *Arg1* | F | ACATTGGCTTGCGAGACGTA |
|  | R | ATCACCTTGCCAATCCCCAG |
| *Cd163* | F | TGCTGTCACTAACGCTCCTG |
|  | R | TCATTCATGCTCCAGCCGTT |
| *β-actin* | F | GTGACGTTGACATCCGTAAAGA |
|  | R | GCCGGACTCATCGTACTCC |
| *Runx2* | F | GCCACCTTTACCTACACCCC |
|  | R | ACTCTGGCTTTGGGAAGAGC |
| *Sp7* (*Osterix*) | F | TTTCTGCGGCAAGAGGTTCA |
|  | R | TTGCTCAAGTGGTCGCTTCT |
| *Ibsp* | F | CACCGTTGAGTATGGGGGAG |
|  | R | TAAGCTCGGTAAGTGTCGCC |
| *Ocn* | F | TGAACAGACTCCGGCGCTAC |
|  | R | AGGGCAGCACAGGTCCTAA |
| *RUNX2* | F | GCGCATTCCTCATCCCAGTA |
|  | R | GGCTCAGGTAGGAGGGGTAA |
| *SP7* (*OSTERIX)* | F | TCTGCGGGACTCAACAACTC |
|  | R | TAGCATAGCCTGAGGTGGGT |
| *IBSP* | F | GACACCACAGAGACCGGAAG |
|  | R | CCAAAAGGTGGGGAAGTGGT |
| *OCN* | F | CACACTCCTCGCCCTATTG |
|  | R | GGTCTCTTCACTACCTCGCT |

| **Table S2** | |  |
| --- | --- | --- |
| **siRNA sequences used for gene knockdown.** | | |
| **Name** | **F/R** | **Sequences** |
| si-CPT1A #1 | F | GCCAGACGAAGAACAUCGUTT |
|  | R | ACGAUGUUCUUCGUCUGGCTT |
| si-CPT1A #2 | F | CUGCAGCUCGCACAUUACATT |
|  | R | UGUAAUGUGCGAGCUGCAGTT |
| si-CPT1A #3 | F | GCUAUGGUGUUUCCUACAUTT |
|  | R | AUGUAGGAAACACCAUAGCTT |
| si-NQO1 #1 | F | GCCCAUUCAGAGAAGACAUTT |
|  | R | AUGUCUUCUCUGAAUGGGCTT |
| si-NQO1 #2 | F | CCGAGUCAUCUCUAGCAUATT |
|  | R | UAUGCUAGAGAUGACUCGGTT |
| si-AhR #1 | F | GAGGGAUUAACUUCUAGAUTT |
|  | R | AUCUAGAAGUUAAUCCCUCTT |
