# Supplementary material for: Tryptophan-Enriched Lactobacillus rhamnosus GG-derived Nanovesicles Promote Alveolar Bone Regeneration through Macrophage Fatty Acid Oxidation
Source: Biomater Res. 2026 Jul 17;30:0370. doi: 10.34133/bmr.0370 (PMC13376381; doi:10.34133/bmr.0370)
Supplement: Supplementary 1 — Graphical Abstract Figs. S1 and S2 Tables S1 and S2 [file bmr.0370.f1.zip › supplementary figs.docx]

**
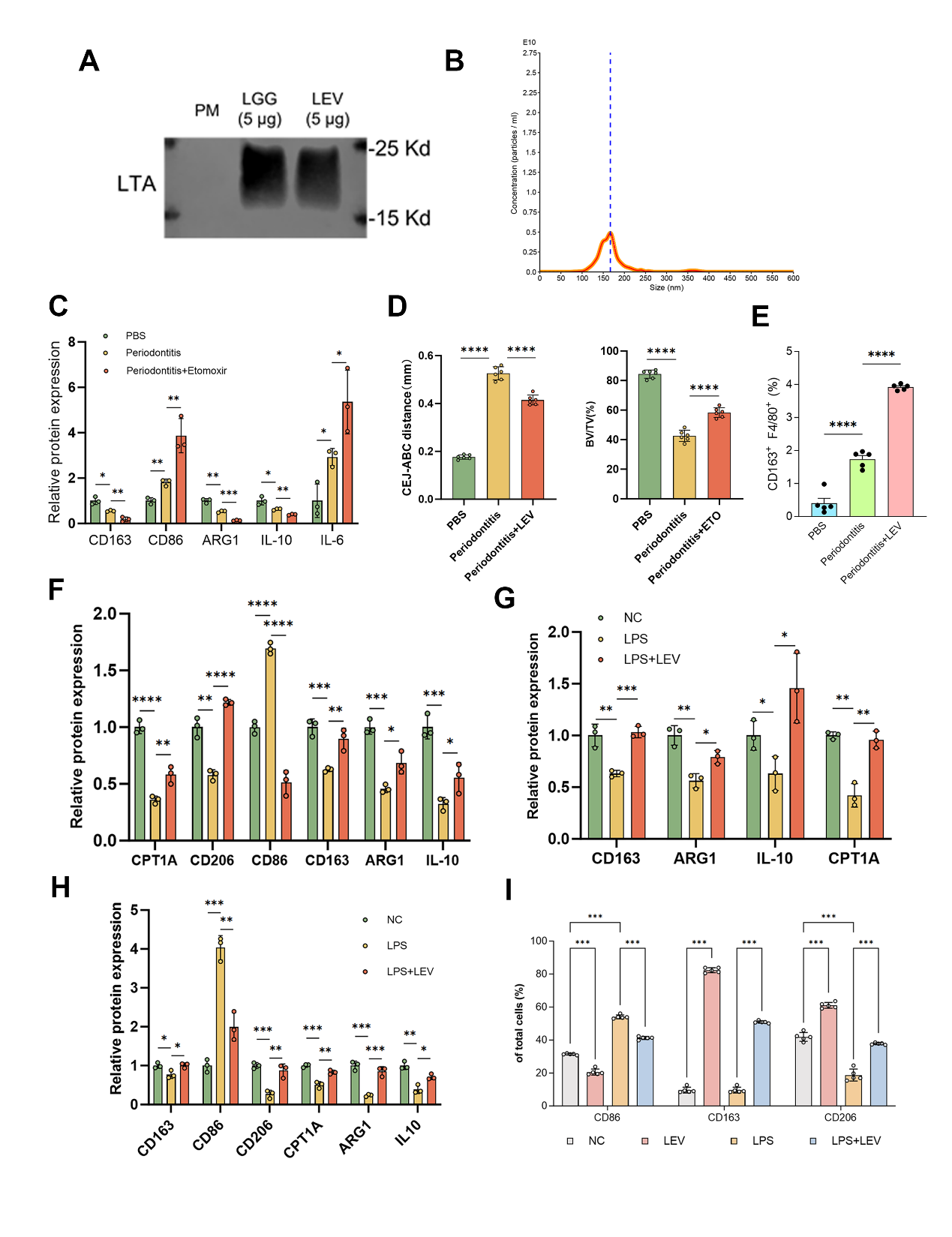
**

**Supplementary Fig. S1**

**(A)** Western blot analysis of LTAexpression in different experimental groups. PM: processed medium control (sterile, uninoculated MRS medium subjected to the identical isolation protocol). **(B)** NTA of LEV concentration following treatment with 0.1 % Triton X-100 at 37 °C for 30 min. The Y-axis scale represents particle concentration in 10^10^ particles/mL. **(C)** Statistical quantification of the relative protein levels of CD163, CD86, ARG1, IL-10, and IL-6 in the PBS, Periodontitis, and Periodontitis+Etomoxir groups as determined by Western blot. **(D)** The distance of the CEJ-ABC in mm and the percentage of BV/TV were analyzed. BV/TV, which represents bone volume fraction, stands for Bone Volume over Tissue Volume. **(E)** The percentage of CD163^+^ F4/80^+^ cells in the gingiva of mice were quantified by flow cytometry. **(F)** CPT1A, CD206, CD86, CD163, ARG1 and IL-10 protein levels relative to that of β-actin were assessed by densitometric analysis. **(G)** CD163, ARG1, IL-10 and CPT1A protein levels relative to that of β-actin were assessed by densitometric analysis. **(H)** CD163, CD86, CD206, CPT1A, ARG1 and IL-10 protein levels relative to that of β-actin were assessed by densitometric analysis. **(I)** The percentage of CD86^+^ or CD163^+^ or CD206^+^ cells were quantified by flow cytometry. **P* < 0.05, ***P* < 0.01, ****P*＜0.001, *****P*＜0.0001 in the indicated groups


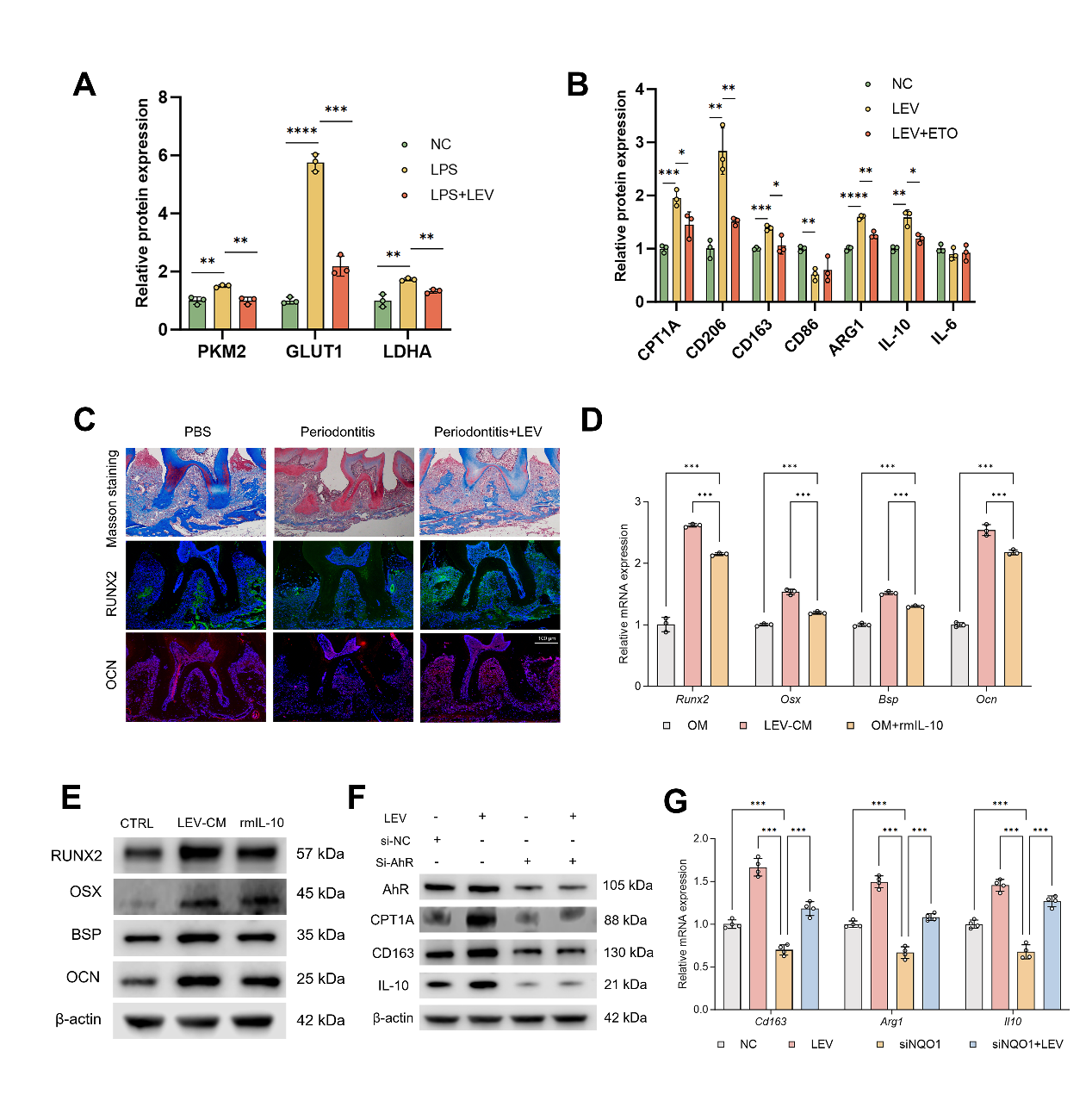


**Supplementary Fig. S2**

**(A)** PKM2, GLUT1 and LDHA protein levels relative to that of β-actin were assessed by quantitative analysis. **(B)** CPT1A, CD206, CD163, CD86, ARG1, IL-10 and IL-6 protein levels relative to that of β-actin were assessed by quantitative analysis. **(C)** Representative histological and immunofluorescence images of the mouse maxilla in the PBS, Periodontitis, and Periodontitis+LEV groups. Top row: Masson's trichrome staining for collagen matrix evaluation. Middle row: Immunofluorescence staining of RUNX2 (green). Bottom row: Immunofluorescence staining of OCN (red). Cell nuclei were counterstained with DAPI (blue). **(D)** The mRNA expression levels of osteogenic markers in osteoblasts treated with standard OM (Ctrl), LEV-CM, or OM+rmIL-10, as analyzed by RT-qPCR. **(E)** Western blot images and corresponding quantitative analysis of osteogenic marker proteins in osteoblasts subjected to the indicated treatments. **(F)** Representative Western blot images showing the protein levels of AhR, CPT1A, CD163, and IL-10 in primary BMDMs transfected with NC-siRNA or Ahr-siRNA followed by LEV treatment. **(G)** The relative gene expression levels of *Cd163*, *Arg1* and *Il-10* were determined by qPCR. **P* < 0.05, ***P* < 0.01, ****P*＜0.001, *****P*＜0.0001 in the indicated groups

**Supplementary Table S1**

Sequences of Primers Used for RT-qPCR. This table lists the forward and reverse primer sequences for each gene analyzed by quantitative real-time PCR in this study.

**Supplementary Table S2**

Sequences of siRNAs Targeting CPT1A and NQO1. This table provides the specific nucleotide sequences of siRNAs designed and constructed for targeting CPT1A and NQO1.
