# Supplementary figures and images for: Tryptophan-Enriched Lactobacillus rhamnosus GG-derived Nanovesicles Promote Alveolar Bone Regeneration through Macrophage Fatty Acid Oxidation
Source: Biomater Res. 2026 Jul 17;30:0370. doi: 10.34133/bmr.0370 (PMC13376381; doi:10.34133/bmr.0370)

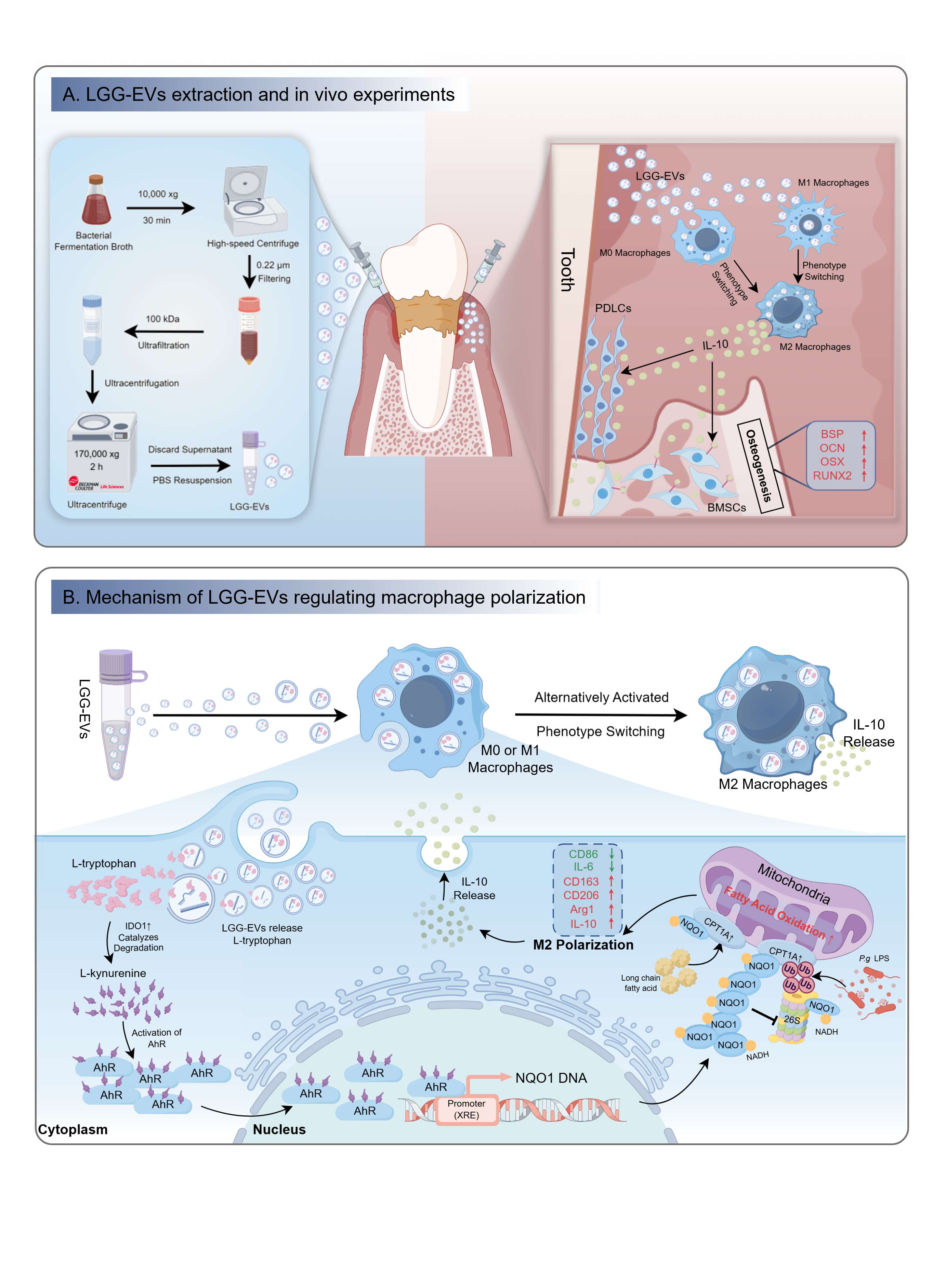

Supplement: Supplementary 1 — Graphical Abstract Figs. S1 and S2 Tables S1 and S2 [file bmr.0370.f1.zip › Graphic Abstract.tif]

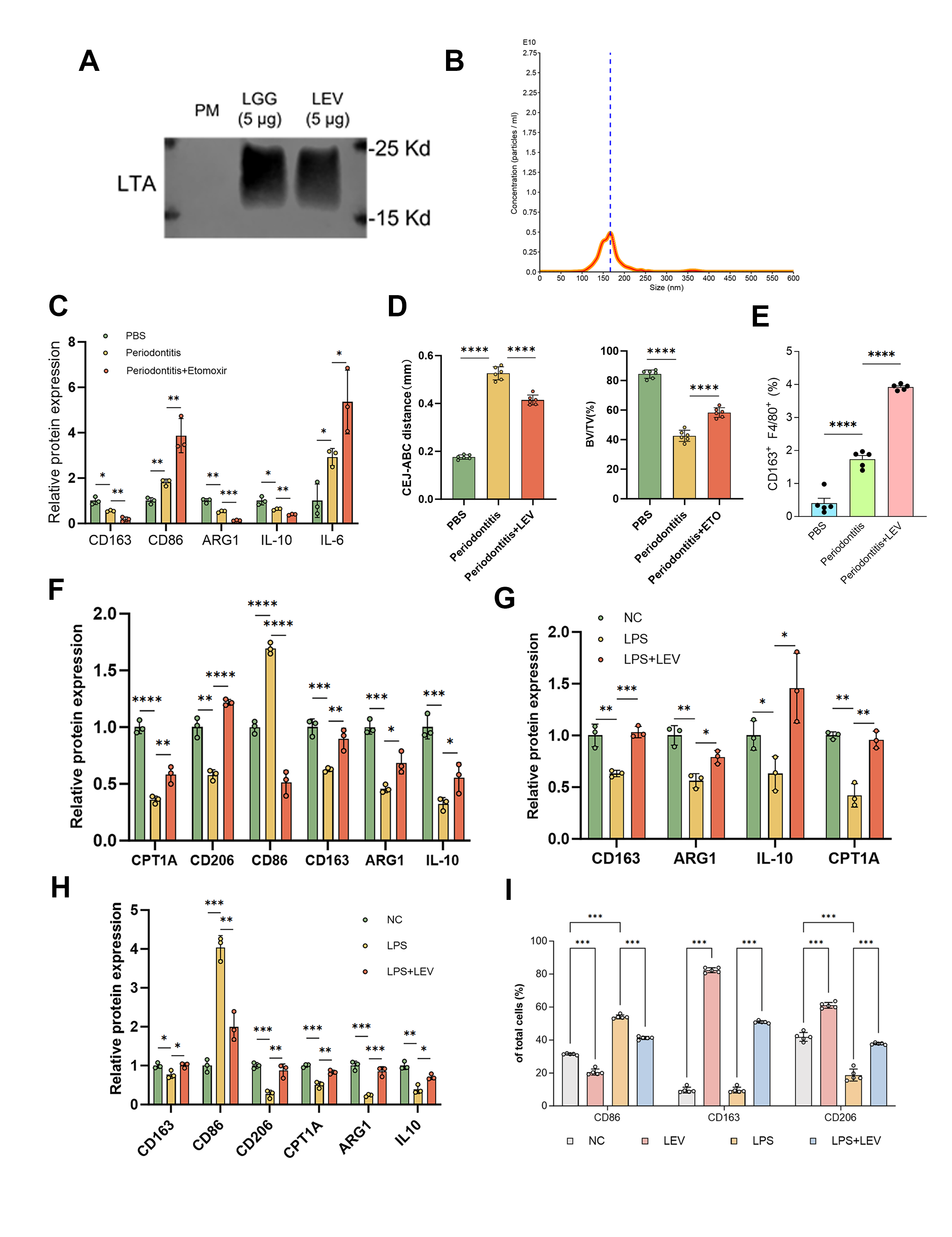

Supplement: Supplementary 1 — Graphical Abstract Figs. S1 and S2 Tables S1 and S2 [file bmr.0370.f1.zip › S1.tif]

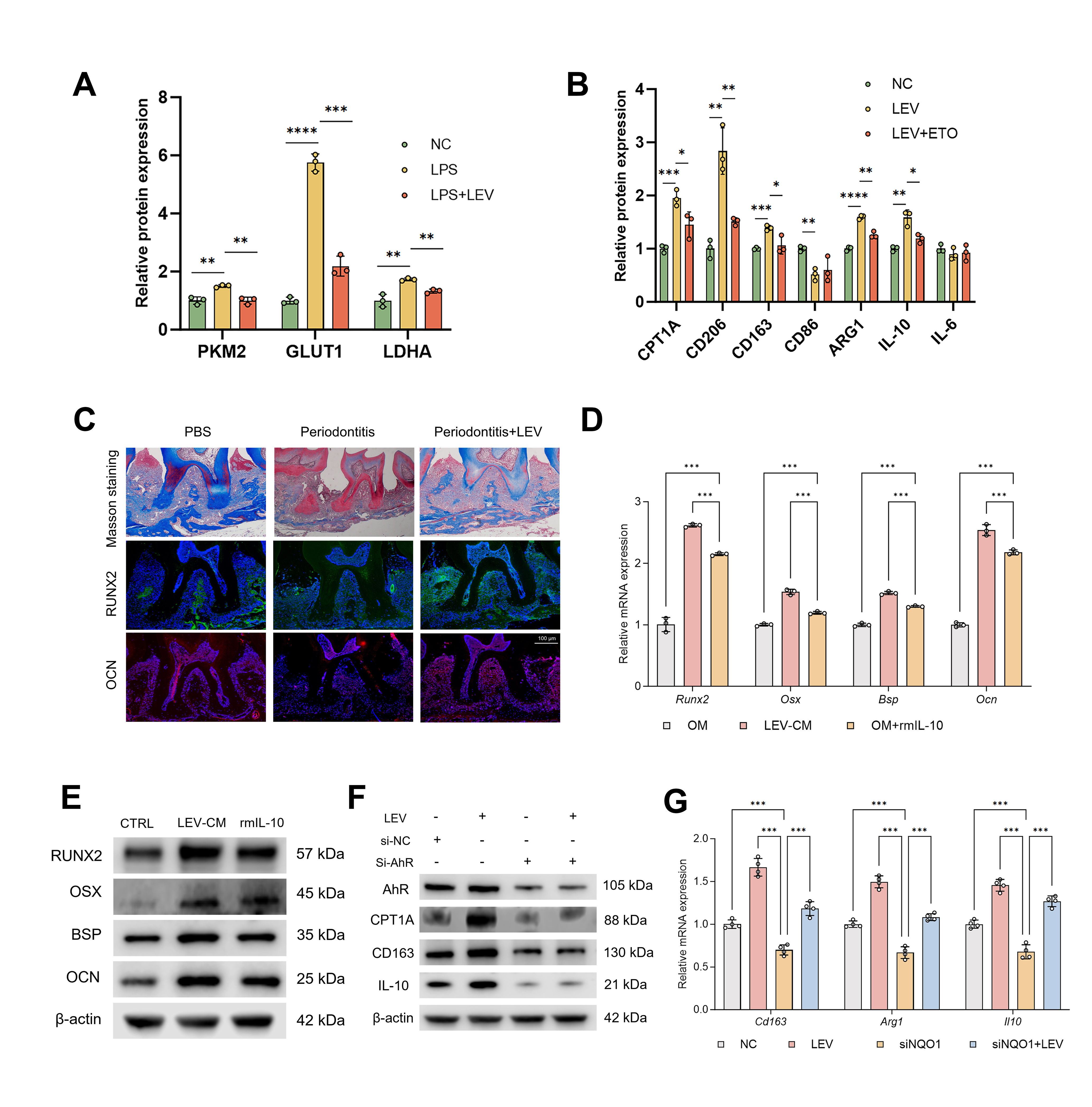

Supplement: Supplementary 1 — Graphical Abstract Figs. S1 and S2 Tables S1 and S2 [file bmr.0370.f1.zip › S2.tif]
